# Quadrupia provides a comprehensive catalog of G-quadruplexes across genomes from the tree of life

**DOI:** 10.1101/gr.279790.124

**Published:** 2025-11

**Authors:** Nikol Chantzi, Akshatha Nayak, Fotis A. Baltoumas, Eleni Aplakidou, Shiau Wei Liew, Jesslyn Elvaretta Galuh, Michail Patsakis, Austin Montgomery, Camille Moeckel, Ioannis Mouratidis, Saiful Arefeen Sazed, Wilfried Guiblet, Panagiotis Karmiris-Obratański, Guliang Wang, Apostolos Zaravinos, Karen M. Vasquez, Chun Kit Kwok, Georgios A. Pavlopoulos, Ilias Georgakopoulos-Soares

**Affiliations:** 1 Institute for Personalized Medicine, Department of Biochemistry and Molecular Biology, The Pennsylvania State University College of Medicine, Hershey, Pennsylvania 17033, USA;; 2 Institute for Fundamental Biomedical Research, BSRC “Alexander Fleming,” Vari 16672, Greece;; 3 Department of Basic Sciences, School of Medicine, University of Crete, Heraklion 71003, Greece;; 4 Department of Chemistry and State Key Laboratory of Marine Environmental Health, City University of Hong Kong, Kowloon Tong, Hong Kong SAR 999077, China;; 5 Shenzhen Research Institute of the City University of Hong Kong, Shenzhen 518057, China;; 6 Advanced Biomedical Computational Science, Frederick National Laboratory for Cancer Research, Frederick, Maryland 21702, USA;; 7 Advanced Manufacturing Laboratory, Department of Manufacturing Systems, Faculty of Mechanical Engineering and Robotics, AGH University of Krakow, Krakow 30-059, Poland;; 8 Division of Pharmacology and Toxicology, College of Pharmacy, The University of Texas at Austin, Dell Pediatric Research Institute, Austin, Texas 78723, USA;; 9 Department of Life Sciences, School of Sciences, European University Cyprus, Nicosia 1516, Cyprus;; 10 Cancer Genetics, Genomics and Systems Biology Laboratory, Basic and Translational Cancer Research Center (BTCRC), Nicosia 1516, Cyprus

## Abstract

G-quadruplex DNA structures exhibit a profound influence on essential biological processes, including transcription, replication, telomere maintenance, and genomic stability. These structures have demonstrably shaped organismal evolution. However, a comprehensive, organism-wide G-quadruplex map encompassing the diversity of life has remained elusive. Here, we introduce Quadrupia, the most extensive and well-characterized G-quadruplex database to date, facilitating the exploration of G-quadruplex structures across the evolutionary spectrum. Quadrupia has identified G-quadruplex sequences in 108,449 reference genomes, with a total of 140,181,277 G-quadruplexes. The database also hosts a collection of 319,784 G-quadruplex clusters of 20 or more members, annotated by taxonomic distributions, multiple sequence alignments, profile hidden Markov models, and cross-references to G-quadruplex 3D structures. Examination of G-quadruplexes across functional genomic elements in different taxa indicates preferential orientation and positioning, with significant differences between individual taxonomic groups. For example, we find that G-quadruplexes in bacteria with a single replication origin display profound preference for the leading orientation. Finally, we experimentally validate the most frequently observed G-quadruplexes using CD-spectroscopy, UV melting, and fluorescent-based approaches.

DNA G-quadruplexes (G4s) are one of the most thoroughly studied non-B DNA structures. G4s are commonly found in GC-rich areas of a genome and are characterized by Hoogsteen base pairs, in which hydrogen bonds link four guanine bases into a square planar formation known as a G-quartet. Multiple G-quartets, stacked on top of each other, lead to the formation of G4 structures (Fig. 1A; Spiegel et al. 2020). Their presence and formation in telomeric regions provided early evidence that G4s are a native DNA conformation (Parkinson et al. 2002). Furthermore, an array of studies have shown that G4s are involved in processes such as gene regulation (Huppert et al. 2008; Brooks et al. 2010; Hänsel-Hertsch et al. 2016; Lee et al. 2020; Lago et al. 2021; Robinson et al. 2021; Shen et al. 2021; Spiegel et al. 2021; Georgakopoulos-Soares et al. 2022a,b; Li et al. 2023), alternative splicing modulation (Huang et al. 2017; Georgakopoulos-Soares et al. 2022c; Ghosh et al. 2023), 3D genome structure organization (Li et al. 2021; Wulfridge et al. 2023; Yuan et al. 2023), and translation (Kumari et al. 2007; Bugaut and Balasubramanian 2012; Song et al. 2016; Murat et al. 2018; Lyu et al. 2021; Georgakopoulos-Soares et al. 2022b), among other functions. Additionally, G4s are linked to genomic instability and have been associated with several diseases, including cancer and neurodegenerative disorders (Simone et al. 2015; Georgakopoulos-Soares et al. 2018; Wang et al. 2021; Makova and Weissensteiner 2023; Wang and Vasquez 2023; Zhang et al. 2023).

**Figure 1.**
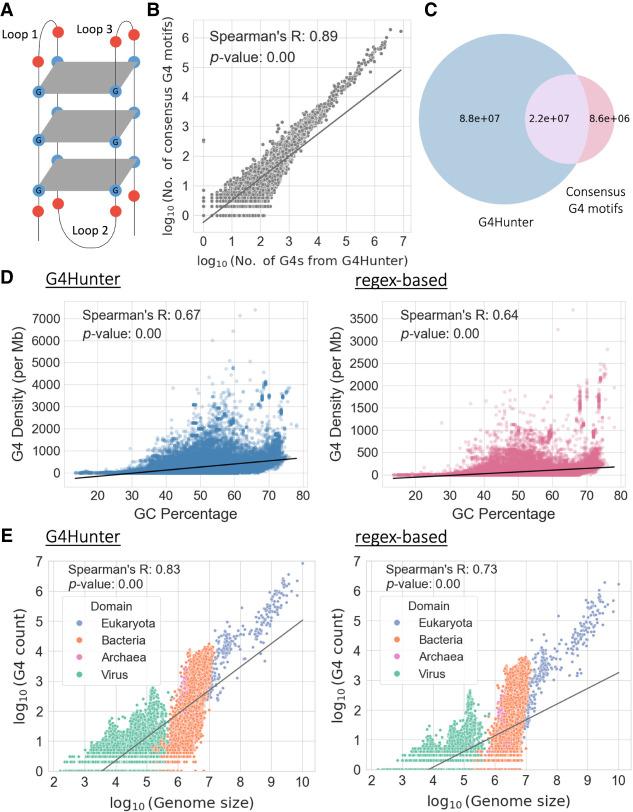
Characterization of G-quadruplexes (G4s) across 108,534 genomes. (*A*) Schematic illustration of a G4. (*B*) Scatter plot displaying the association between the number of G4s detected per species using G4Hunter (*x*-axis) and the regex-based (*y*-axis) algorithms. Values on both axes are represented in log_10_ scale. (*C*) Venn diagram showing the number of shared G4s found by the two methods. The blue circle represents the total number of G4s detected across all species using the G4Hunter method; the red circle represents the total number of G4 motifs detected using the regex-based algorithm across all species; and the overlapping purple region represents the G4s found using both methods. (*D*) Association between GC percentage and the number of G4s observed per million base pairs in each genome, based on G4s from G4Hunter (*left*) and G4 motifs detected from the regex-based algorithm (*right*). (*E*) Association between the length of the genome and the number of G4s detected, based on G4s from G4Hunter (*left*) and G4 motifs obtained from the regex-based algorithm (*right*). Values on both axes are represented in log_10_ scale. Each dot represents an organismal genome, and the color represents the taxonomic subdivision among the three domains of life and viruses that the organism belongs to. The lines in *B*, *D*, and *E* represent regression lines for the respective associations.

There are several established methods for detecting G4s in silico, in vitro and in vivo and characterizing their formation kinetics and stability (Kwok and Merrick 2017). There are also efforts to develop in silico methods that identify and annotate quadruplexes in the atomic data of nucleic acid structures, such as ElTetrado and DSSR (Lu et al. 2015; Zok et al. 2020; Adamczyk et al. 2023). In vitro approaches include chemical and biophysical methods such as nuclear magnetic resonance (NMR) (Ida and Wu 2008; Harkness and Mittermaier 2017), circular dichroism (CD) (Del Villar-Guerra et al. 2018; Chan et al. 2019), and UV thermal melting (Mergny and Lacroix 2009). G4-sequencing methods (G4-seq and rG4-seq) use the property of stable G4 structures to impede DNA and RNA polymerase progression in vitro, enabling genome-wide or transcriptome-wide high-resolution detection of putative DNA and RNA G4s through high-throughput sequencing (Chambers et al. 2015; Kwok et al. 2016; Marsico et al. 2019; Zhao et al. 2022). To detect G4s in vivo, immunostaining with antibodies specifically designed against G4s (Biffi et al. 2013) and ChIP-seq genome-wide approaches have been implemented (Hänsel-Hertsch et al. 2016). Imaging of G4 formation has been demonstrated in live cells using fluorescence imaging (Di Antonio et al. 2020; Summers et al. 2021). Such experimental approaches have enabled the derivation of patterns enabling the prediction of sequences that can adopt G4 DNA structures.

Early computational studies showed that conserved sequence motifs can accurately capture a significant proportion of potential G4-forming regions (Huppert and Balasubramanian 2005). Since then, the consensus G4 motif, G ≥ 3N1–7G ≥ 3N1–7G ≥ 3N1–7G ≥ 3, has been used to quantify the number and distribution of G4s and is utilized in numerous available methods and studies (Kikin et al. 2006; Huppert and Balasubramanian 2007; Zhang et al. 2008; Wong et al. 2010; Cer et al. 2013). However, it has become evident that certain G4s do not conform to this consensus motif. Thus, additional computational approaches have since been implemented to capture a broader range of putative G4-forming sequences (Eddy and Maizels 2006; Kikin et al. 2006; Bedrat et al. 2016; Dhapola and Chowdhury 2016; Garant et al. 2017; Sahakyan et al. 2017; Di Salvo et al. 2018; Belmonte-Reche and Morales 2020; Lombardi and Londoño-Vallejo 2020; Miskiewicz et al. 2021).

Previous work has characterized the distribution and frequency of G4s in species from multiple taxonomies. In eukaryotes, G4s are enriched at *cis*-regulatory elements (Lago et al. 2021; Shen et al. 2021; Spiegel et al. 2021; Georgakopoulos-Soares et al. 2022a,d), including in promoters, enhancers, and CTCF binding sites, whereas in higher eukaryotes, they have also emerged in proximity to splice sites to modulate alternative splicing (Georgakopoulos-Soares et al. 2022c). In bacteria, an analysis of 1627 genomes revealed significant differences in the frequencies of G4s, with Deinococcota displaying the highest density in their genome (Bartas et al. 2019), whereas another study revealed enrichment of G4s in functional elements when examining eighteen prokaryotic genomes (Rawal et al. 2006). When investigating the genomes of archaea and viral species, large differences in the frequencies and functions of G4s were also reported (Métifiot et al. 2014; Lavezzo et al. 2018; Saranathan and Vivekanandan 2019; Brázda et al. 2020).

There are multiple databases focusing on the structure formation of different G4 sequences, the interactions of G4s with proteins, and individual genes. G4IPDB is a database dedicated to proteins that interact with G4-forming nucleic acid sequences (Mishra et al. 2016), and DSSR-G4DB is a database of G4s in the Protein Data Bank (PDB), in which DSSR (Lu et al. 2015) was employed to analyze the spatial structure of G4s (Lu 2020). Greglist is a database of G4 regulated genes (Zhang et al. 2008), and GRSDB2 and GRS_UTRdb are databases on G4s in their roles in regulation of gene expression in pre-mRNAs and mRNAs (Kikin et al. 2008).

To date, there are different databases available incorporating the identification of potential G4 DNA-forming sequences in different organisms; however, all available data sets cover a small number of species or taxa. Zhong et al. (2023) constructed G4Bank, a G4 database containing 6 million G4s across 13 species (Zhong et al. 2023), whereas GAIA currently harbors G4s for 61 different organismal genomes (Vannutelli et al. 2023). Another database named G4Atlas contains only 238 experimental G4s from 10 species (Yu et al. 2023); ProQuad contains quadruplex information of 146 prokaryotes (Yadav et al. 2007); G4-virus is a database of G4s locations in the human viruses’ genomes (Lavezzo et al. 2018); and Plant-GQ harbors G4s across 195 plants (Ge et al. 2019). Genome-wide G4-seq-based detection of G4s was performed in 19 organisms (Chambers et al. 2015; Marsico et al. 2019) and rG4-seq maps in the genomes of *Pseudomonas aeruginosa* and *Escherichia coli* (Shao et al. 2020). Other databases are focused only on a small set of species or are providing a set of experimentally validated G4s (Li et al. 2013; Ghosh et al. 2021; Wang et al. 2022; Zok et al. 2022; Qian et al. 2024), yet none of these databases contain a wide range of genomes, representing species across the taxonomic subdivisions in the tree of life. Thus, there is a gap in extensive evolutionary research on G4s as there is a lack of genome-wide maps of predicted G4s across all species with a reference genome available.

Here, we present “Quadrupia” (https://www.pavlopoulos-lab.org/quadrupia), the largest and, to our knowledge, most comprehensive database of putative G4 DNA-forming sequences to date, covering 108,534 species and encompassing 87,160,084 putative G4 DNA-forming sequences. The experimental validation of highly prevalent G4s across the tree of life suggests that our predictions in the database are accurate. We identify clusters of G4 DNA-forming sequences based on sequence identity and characterize the preference of each cluster in different taxonomies. We also perform G4 DNA analyses across the taxonomic subdivisions and at individual species, observing marked differences in G4 DNA density and distribution across functional genomic elements. For example, we discovered that G4s are strongly enriched in the leading strand relative to the lagging strand orientation in bacterial genomes and are enriched in a subset of splice junctions in specific phyla. We expect that our work will open opportunities for researchers to delve into the functional, regulatory, and evolutionary origin of G4 DNA, using the plethora of available complete organismal genomes.

## Results

### Identification of potential G4-forming sequences in organismal genomes across the tree of life

We examined 108,459 organismal reference genomes spanning the three domains of life and viruses and identified putative G4 DNA-forming sequences in each of them using two methods, namely, the regex-based algorithm and the predicted G4s from the state-of-the-art G4 detection method G4Hunter (Bedrat et al. 2016). First, we investigated the percentage of G4s identified with these two algorithms that were also detected in G4-seq data for seven species (Supplemental Fig. 1; Marsico et al. 2019). We report that the percentage of G4s detected with G4Hunter that were also identified with G4-seq ranged between 44.81% and 80.56%, whereas for the regex-based algorithm they ranged between 65.15% and 95.87% (Supplemental Fig. 1). For both computational methodologies, we observe high concordance between the experimentally validated and computationally derived G4s across all examined model organisms. Next, we scanned the 108,459 organismal reference genomes for G4s using the regex-based algorithm and G4Hunter; we find an average of 279.43 G4s per genome with the regex-based G4 motif detection method and 1,013.05 per genome with the G4Hunter-based method, observing a high degree of concordance between the number of G4s detected with each method for each genome (Spearman's correlation r = 0.89, *P*-value = 0) (Fig. 1B,C; Supplemental Fig. 2A). G4 sequences found using the regex-based algorithm and the G4Hunter algorithm that have at least a 50% overlap were considered common G4 sequences found using both methods (Fig. 1C).

G4s are highly GC-rich sequences; we therefore investigated the association between each organismal genome's GC content and the number of G4s detected. We observe a strong correlation between the density of G4s and the GC content of each species, with both G4 detection approaches (Spearman's correlations r = 0.67, *P*-value = 0 for results from G4Hunter; r = 0.64, *P*-value = 0 for the regex-based algorithm) (Fig. 1D). We also find a large disparity in the G4 motif density between genomes, for a given GC content, indicating that GC content can only partially account for the G4 motif density differences between organismal genomes (Fig. 1D).

Next, we investigated the number of G4s observed across the taxonomies as a function of genome size. As expected, larger genomes harbored a larger number of G4s, with eukaryotes and viruses having the highest and lowest number of G4s, respectively (Fig. 1E). We also find that there is a large dispersion within the domains of life and viruses (Fig. 1E). We report that the species with the highest G4 motif density in their genomes based on both approaches are *Grapevine fleck virus* and *Alcea yellow mosaic virus*, with densities of 104.57 and 93.27 G4s per kilobase, respectively, based on the regex-based algorithm, and densities of 438.92 and 371.51 G4s per kilobase, respectively, based on G4Hunter. We also examined the association between the proportion of the genome covered by genes and the genomic density of G4s per genome and observed a weak negative correlation in the case of eukaryotes (Spearman's correlations r = −0.25, *P*-value = 0 for results from G4Hunter; r = −0.34, *P*-value = 0 for the regex-based algorithm (Supplemental Fig. 2B).

### Characterization of G4s across taxonomic subdivisions

**Figure 2.**
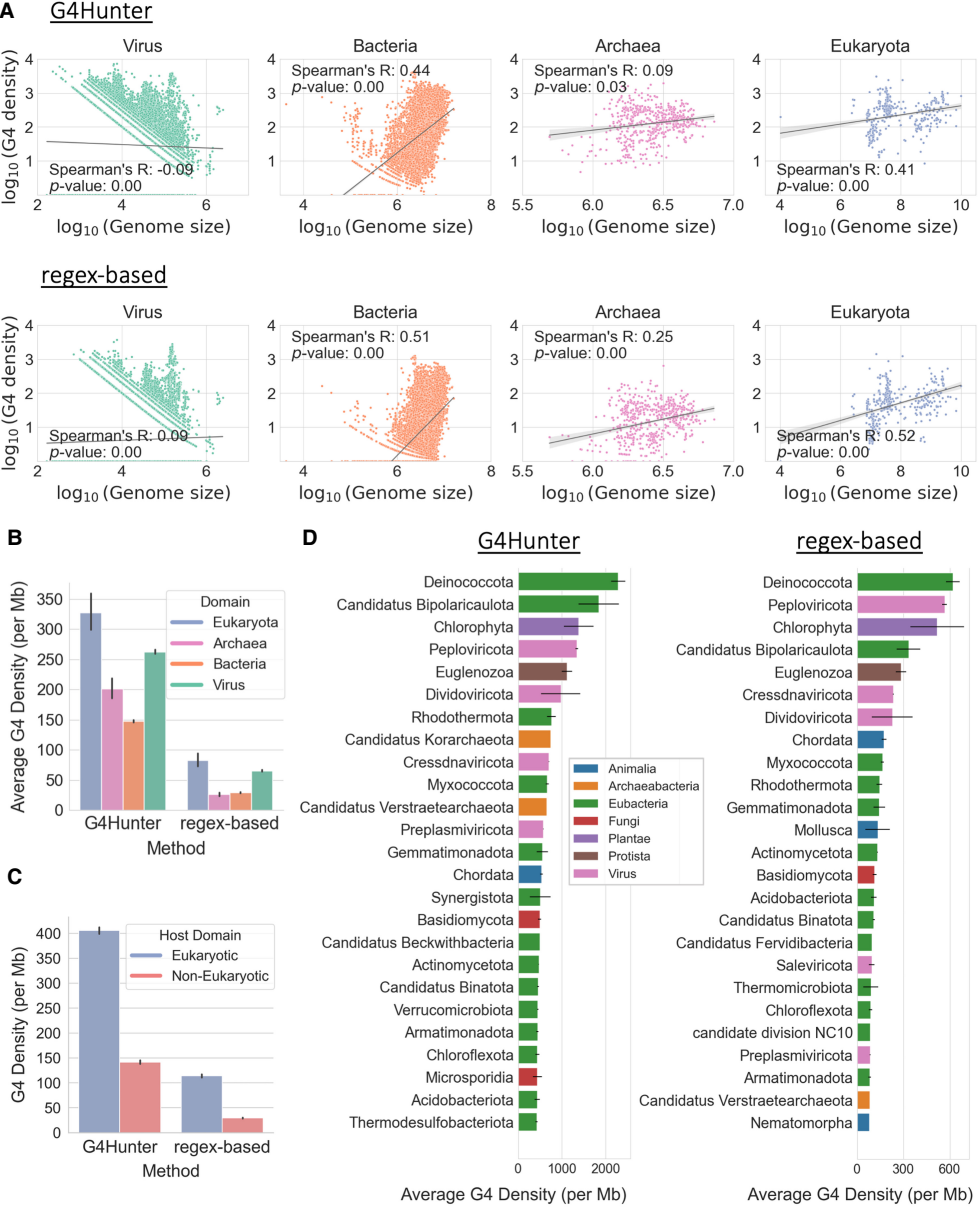
Taxonomic characterization of G4s across the tree of life. (*A*) Association between genome size and average G4 motif density (per million bases), categorized by the taxonomic subdivision among the three domains of life and viruses to which the organism belongs. Each dot represents an organismal genome, and the lines in each subplot represent the regression line for the respective associations. Values on both axes are represented in log_10_ scale. The subplots are based on G4s from G4Hunter (*top* row) and from the regex-based algorithm (*bottom* row). (*B*) Average density of G4s (per million bases) for organisms belonging to each domain of life and viruses. The error bars represent 95% confidence intervals. (*C*) Average density of G4s (per million bases) for viral genomes, categorized by their host domain (eukaryotic or prokaryotic). The error bars represent 95% confidence intervals. (*D*) Average density of G4s (per million bases) of organisms belonging to each phylum, based on G4s from G4Hunter (*left*) and G4 motifs detected using the regex-based algorithm (*right*). The plots include the top 25 phyla having the highest average G4 motif densities. The error bars represent SEM.

Next, we investigated differences in the distribution and density of G4s between taxonomic subgroups. We first examined the association between the genome size and the density of G4s for organisms in the three domains of life and viruses. We find that there is a moderately positive correlation between the density of G4s and the genome size in bacteria (Spearman's correlations r = 0.44, *P*-value = 0 for results from G4Hunter; r = 0.51, *P*-value = 0 for regex-based method) and eukaryotes (Spearman's correlations r = 0.41, *P*-value = 0 for results from G4Hunter; r = 0.52, *P*-value = 0 for regex-based method). In archaea, results from G4Hunter show no correlation, whereas results from the regex-based method show a very weak positive correlation (r = 0.25, *P*-value = 0). In viruses, no correlation between the density of G4s and the genome size is observed in results from either method (Fig. 2A; Supplemental Table 1). We also examined the G4 motif density in the three domains of life and viruses. We report that the highest and lowest density of G4s is observed in eukaryotes (327.03 G4s per megabase) and bacteria (147.81 G4s per megabase), respectively, based on the results from G4Hunter. However, based on the G4 motifs derived from the regex-based algorithm, the highest and lowest densities of G4s are observed in eukaryotes (82.76 G4s per megabase) and archaea (26.40 G4s per megabase) (Fig. 2B; Supplemental Table 1).

We further subdivided this analysis at the phylum level. The average G4 motif density varied between phyla belonging to each domain, with about 2285-fold variation in bacteria, 1382-fold in eukaryotes, 1336-fold in viruses, and 742-fold in archaea. We observe that the top five phyla with the highest G4 motif density are Deinococcota, Candidatus Bipolaricaulota, Chlorophyta*,* Peploviricota, and Euglenozoa for both the G4Hunter and the regex-based algorithms (Fig. 2D), indicating strong concordance between the two G4 detection approaches used. Additionally, the phyla with the highest G4 motif density belong to different domains of life and viruses and to different kingdoms (Fig. 2D; Supplemental Fig. 4; Supplemental Table 1), highlighting the significant variability in G4 motif density between phyla belonging to the same taxonomic supergroup. These findings underscore the profound differences in G4 motif density in organismal genomes across the tree of life.

Previous studies have shown a correlation between G4 sequence composition in viruses and their hosts (Li et al. 2022). We therefore investigated if the viruses with the highest G4 motif densities also have hosts with a high G4 motif density. Out of the 63,658 viral genomes that we analyzed in this study, we selected the top 100 viral genomes with the highest G4 motif density. The majority of the viruses were found to be part of the phylum Peploviricota (73% in G4Hunter results and 94% in results obtained from G4 motifs detected using the regex-based algorithm) having vertebrate hosts, followed by phylum Kitrinoviricota (11% in G4Hunter results and 4% in results obtained from the regex-based algorithm) having plant and fungal hosts. Despite Peploviricota and Kitrinoviricota accounting for only 2.2% and 2.8%, respectively, of all the viral genomes we studied, they collectively constituted a significant proportion of the top 100 viruses with the highest G4 motif density. Notably, both have eukaryotic hosts that we found to have the highest G4 motif density among the three domains of life (Fig. 2B). Further investigating the G4 motif densities of all viral genomes with hosts across the three domains of life, we found viruses with eukaryotic hosts exhibited the highest average G4 motif density (Fig. 2C; Supplemental Tables 1, 2), followed by archaeal and bacterial hosts (Supplemental Fig. 2C). A two-sample *t*-test indicated a statistically significant difference (*P*-value = 0.0 for G4Hunter results; *P*-value = 0.0 for G4 motifs obtained from the regex-based algorithm) in the average G4 motif densities between the viruses with eukaryotic and prokaryotic hosts, with the viruses with eukaryotic hosts having substantially higher G4 motif densities. These results indicate a possible association between the G4 motif densities of viruses and their hosts.

### Genomic distribution of G4s in genomic subcompartments across organismal genomes

G4s are unevenly distributed in the human genome and are enriched in specific subcompartments in which they have functional roles associated with transcription and translation (Huppert et al. 2008; Brooks et al. 2010; Hänsel-Hertsch et al. 2016; Huang et al. 2017; Chen et al. 2018; Guiblet et al. 2021b; Lago et al. 2021; Robinson et al. 2021; Shen et al. 2021; Spiegel et al. 2021; Georgakopoulos-Soares et al. 2022a,c,d; Li et al. 2023). We therefore investigated the extent to which the G4 motif density varied within organismal genomes, in different genomic subcompartments, and if such differences were influenced by the taxonomic group studied.

**Figure 3.**
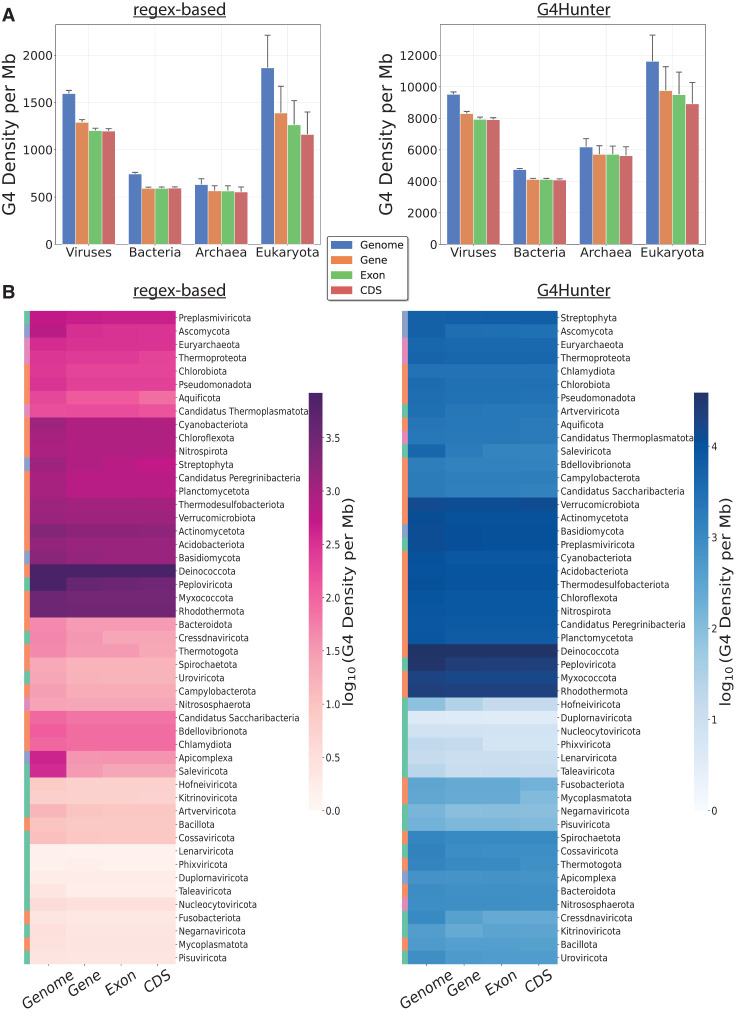
G4 motif density in different genomic subcompartments for organisms across the tree of life. (*A*) Average G4 motif density (per million bases) in genome-wide, genic, exonic, and coding regions, categorized by the taxonomic subdivision among the three domains of life and viruses to which the organism belongs. (*B*) Average G4 motif density (per million bases) in genome-wide, genic, exonic and coding regions, for organisms belonging to different phyla. The colorbar is represented in the log_10_ scale. The subplots in *A* and *B* represent results based on G4s from G4Hunter (*left*) and the regex-based (*right*) algorithms.

To that end, we examined the G4 motif density across the genome and in genic, exonic, and coding regions for each organismal genome across the different taxonomic groups. For the three domains of life and viruses, we observed that the density of G4s is highest when examining the whole genome rather than any specific genomic subcompartment (Fig. 3A). The results were consistent across both methodologies based on G4Hunter results and the results obtained from the regex-based algorithm. Similarly, when examining different phyla based on the results from G4Hunter, genomic-wide G4 distribution has the highest average G4 motif density per megabase. However, there are some notable exceptions. In particular, the eukaryotic phylum Chordata indicates a higher G4 motif density in exonic and coding sequences (CDSs) (Fig. 3B). However, this analysis did not examine the G4 positioning relative to functional genomic sites. Based on G4Hunter methodology, we also find that in eukaryota, the median 24.2% of total genes per species harbor one or more G4s, whereas in archaea 10.1% of total genes have at least one G4 sequence per species, followed by bacteria with 6.6% and viruses with 2.2% (Supplemental Fig. 3). Furthermore, if we partition the genes into the two mutually exclusive sets of protein-coding and noncoding, we find in eukaryotic organisms an even higher median overlap of 25%, whereas across all four domains, the noncoding overlap is lower than its corresponding protein-coding regions (Supplemental Fig. 3). These findings indicate that G4s are highly prevalent in sites associated with transcriptional regulation and transcription.

### Enrichment of G4s relative to transcription start and end sites and splice sites

We next investigated how G4 sequences are distributed concerning transcription start sites (TSSs) and transcription termination sites (TESs). By comparing the varying frequencies of G4s near these sites across different taxonomic groups, we sought to understand potential differences in the regulatory roles of G4s in gene expression among taxa. For G4Hunter-based G4 detection, we observe that there is an enrichment of G4s in the promoter upstream regions for bacteria (1.84-fold enrichment), eukaryotes (1.62-fold enrichment), archaea (1.63-fold enrichment), and viruses (1.54-fold enrichment), whereas similar results were observed from G4s derived using the regular expression-based algorithm (Supplemental Figs. 5, 6). Relative to the TES, we found strong enrichments using the G4Hunter algorithm for archaea (1.57-fold), bacteria (2.18-fold), and viruses (1.77-fold), with weaker enrichment also observed for eukaryotes (1.18-fold), results that were consistent when using the regular expression-based algorithm (Supplemental Figs. 5, 6).

**Figure 4.**
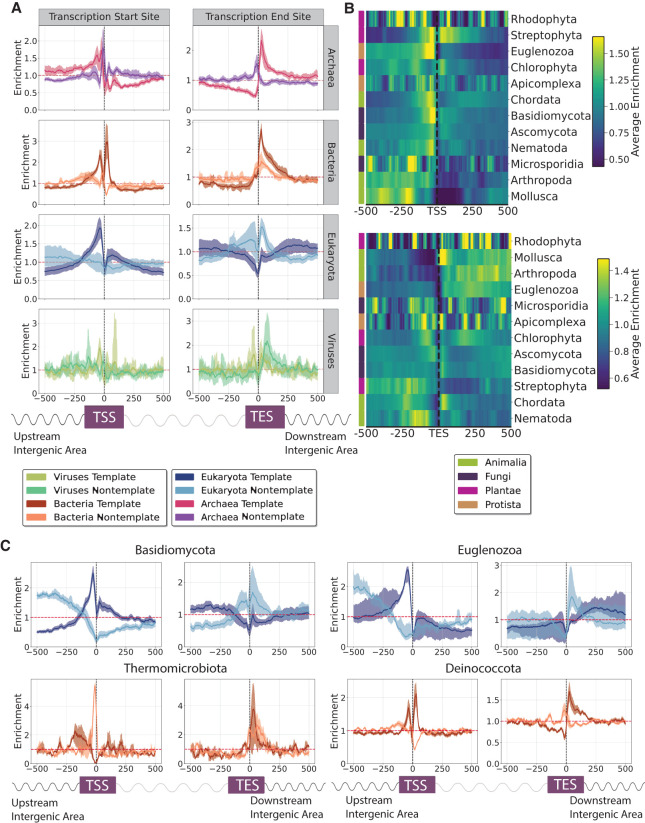
The topography of G4s relative to transcription start sites (TSSs) and transcription end sites (TESs) across the tree of life. (*A*) G4 distribution across the three domains of life and viruses. Results are shown for the template and nontemplate strands separately. (*B*) Distribution of G4s relative to TSSs and TESs across eukaryotic phyla. (*C*) G4 distribution relative to TSSs and TESs for two eukaryotic phyla, Basidiomycota and Euglenozoa, and two bacterial phyla, Thermomicrobiota and Deinococcota. Results are shown for the template and nontemplate strands separately. Error bars represent the 2.5% lowest and 97.5% highest percentile from Monte-Carlo simulations with replacement (N = 1000).

When examining G4s separately in the template and nontemplate strands, we found that there were large differences in their distribution in all domains of life, both relative to the TSSs and TESs (Fig. 4B; Supplemental Fig. 7). For instance, G4 sequences found in bacteria and archaea were predominantly located downstream from the TES on the template strand (enrichments of 2.78-fold and 2.34-fold with the G4Hunter algorithm) relative to the nontemplate strand (enrichments of 1.54-fold and 1.56-fold), whereas G4s on the nontemplate strand were more commonly found in regions preceding the TES (Kolmogorov–Smirnov test, *P*-values < 0.0001, Bonferroni-corrected *P*-values) (Fig. 4B). G4s were depleted in the template strand in the vicinity of the TES, but not in the nontemplate strand (Fig. 4B). We further separated organisms from the three domains of life and viruses into the different phyla. We observe that the distribution of G4s relative to TSSs and TESs are highly variable between the different phyla (Fig. 4B,C; Supplemental Figs. 8, 9).

**Figure 5.**
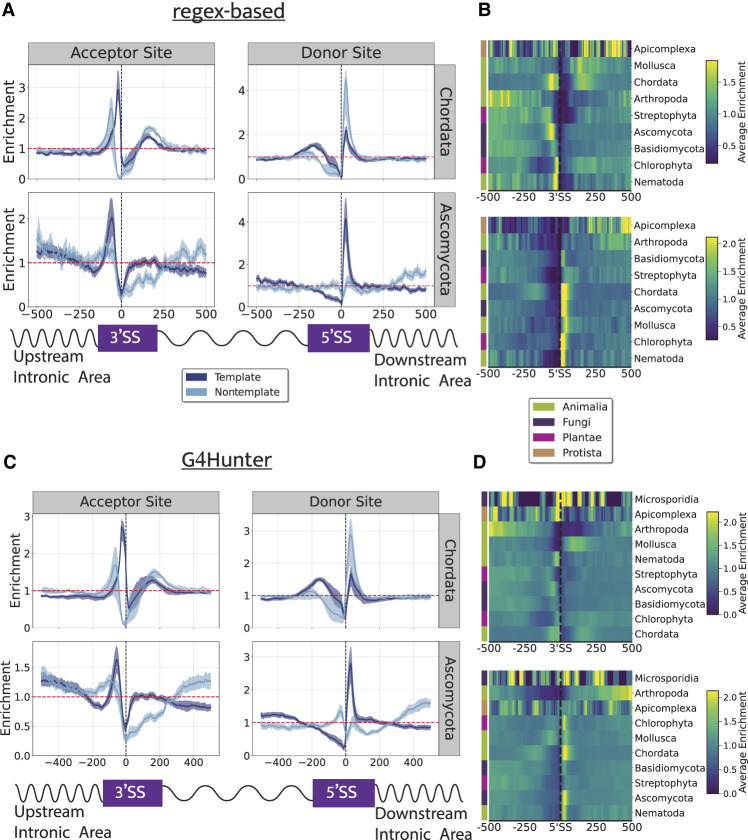
The topography of G4s relative to splice sites in eukaryotic phyla. (*A*) Distribution of G4s relative to 3'ss and 5'ss using G4 motifs derived from the regex algorithm for two eukaryotic phyla, Chordata and Ascomycota. (*B*) Heatmap showing the enrichment of G4s relative to splice sites across different phyla for both the template and the nontemplate strand combined. (*C*) Distribution of G4s relative to 3'ss and 5'ss using G4 motifs derived from the G4Hunter-based algorithm for two eukaryotic phyla, Chordata and Ascomycota. (*D*) Heatmap showing the enrichment of G4s derived from the G4Hunter algorithm relative to splice sites across different phyla for both the template and the nontemplate strand combined. Results are shown for the template and nontemplate strands separately. Confidence intervals in plots *A* and *C* represent the 2.5% lowest and 97.5% highest percentile from Monte-Carlo simulations with replacement (N = 1000).

We next investigated how G4 sequences are distributed concerning splice sites in eukaryotic organisms. By comparing the varying frequencies of G4s near splice sites across different taxonomic groups, we sought to understand potential differences in the regulatory roles of G4s in splicing among taxa. We found that G4s display a remarkable enrichment in the phyla of Chordata and Ascomycota in the intronic area upstream of the acceptor sites and in the intronic area downstream from the donor sites, suggesting that G4s regulate the RNA splicing for these eukaryotic phyla (Fig. 5A,B). For both G4 detection methods, for Chordata we observe that there is roughly 3.3-fold and 2.7-fold enrichment, whereas for Ascomycota, we observed roughly 2.7-fold and 2.1-fold, respectively, enrichment on the template strand in the intron of the upstream splice site (3′ss). For Chordata, in the intronic area of the downstream splice site (5′ss), we observe a 4.2-fold enrichment for G4 motifs on the nontemplate strand derived from the regex algorithm and a 2.1-fold enrichment for the G4Hunter-based method. Furthermore, in the aforementioned phyla, significant differences were observed in the corresponding template and nontemplate distributions (Fig. 5A,B). For instance, G4 sequences found in Chordata were predominantly located in the intron preceding the 3′ss on the template strand, whereas G4s on the nontemplate strand were highly enriched in the intronic area downstream from the 3′ss. In contrast, in Ascomycota the template strand displayed a higher enrichment compared with the nontemplate both upstream of the 3′ss and downstream from the 5′ss (Fig. 5C,D). This indicates that G4s have potential regulatory roles in splicing in Chordata, as previously shown from work in humans (Georgakopoulos-Soares et al. 2022c).

### Differences in the intervening loop length in G4 DNA between taxonomies

As a subsequent step to our analysis, we investigated how the G4s and their associated loop lengths vary across the three domains of life, including viruses. For G4 motifs derived from the regex algorithm, we observe that the total G4 length and loop length distributions of G4s originating from eukaryotic or viral organismal genomes show high variance in contrast to archaea or bacteria that are more centralized (Supplemental Fig. 10B). Moreover, viruses display a preference for large G4 sequences. In G4s extracted with G4Hunter, we observe that these differences are less pronounced, and the distribution of individual taxonomies is more akin to the distribution containing all organismal genomes (Supplemental Fig. 11), indicating that the loop length of G4s varies significantly between taxonomies.

### Patterns of G4s in origins of replication and replication strand polarity

**Figure 6.**
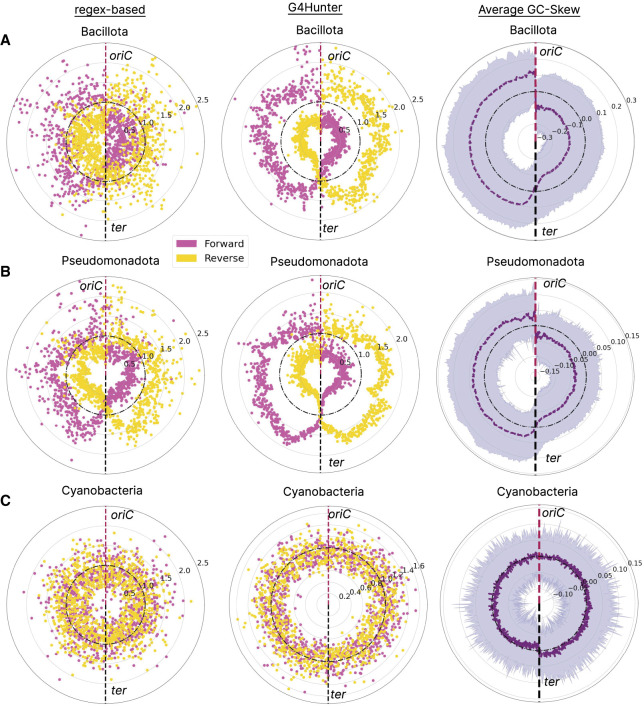
G4 distribution patterns relative to replication origins in bacterial phyla. Results shown for Bacillota (*A*), Pseudomonadota (*B*), and Cyanobacteria (*C*). Enrichment of G4s in forward- and reverse-strand orientation is shown in yellow and pink, respectively. Results are shown for G4 Hunter-based and regex-based algorithms. We discretized the circular bacterial chromosomes in 1001 bins, relative to *oriC*, for each calculating the total number of G4 sequences divided by the total number of G4s that span the whole chromosome, to estimate the local enrichment of G4s. The average GC skew is also calculated and shown in purple.

We investigated if G4s are differentially found in the forward and reverse strands in bacteria, relative to the origin of replication. To achieve this, we generated N = 1000 equal-sized bins per genome, representing the distance from the origin of replication (see Methods). We used only bacteria with a single replication origin, which constitute the vast majority of cases, to perform this analysis. In these, there are two diverging replication forks proceeding from the same origin of replication (*oriC*) in opposite directions until they reach the replication terminator sites (*ter*). We mapped G4s in the forward and reverse strands in each genomic bin and observed highly biased distributions when comparing the relative enrichment between the two strands across the genome (Fig. 6A–C; Supplemental Fig. 11). These biases reflect a preference for G4s in the leading strand and a dearth in the lagging strand. At individual phyla, we observe strong biases in G4 preference in Bacilliota and Pseudomonadota but not in Cyanobacteria (Fig. 6A–C; Supplemental Fig. 11).

To further evaluate these differences, we calculated the average GC skew levels (GC skew = (G − C)/(G + C)) in the different parts of the genomes of each organism in these phyla (Grigoriev 1998). Guanines are more abundant in the leading strand, and therefore, negative GC skew scores are linked to the leading strand (Grigoriev 1998; Merrikh and Merrikh 2018). Our observations indicate that phyla exhibiting pronounced GC skew biases between leading and lagging strands tend to have differences in the levels of G4s between the leading and lagging orientations. Conversely, in phyla such as Cyanobacteria, in which the GC skew is less pronounced, there is less disparity in the G4 frequency between the strands. Consequently, the distribution of G4s between leading and lagging strands in these phyla tends to be more uniform. We conclude that the distribution of G4s across bacterial genomes of specific phyla can be profoundly biased toward the leading strand.

### Examination of G4s in human *cis*-regulatory elements and replication origin sites

Previous studies have found roles for G4s at origins of replication (Besnard et al. 2012; Qiu et al. 2025). We therefore investigated the distribution of G4s relative to sites of human origins of replication using maps of stochastic and core origins of replication (Akerman et al. 2020). Core origins of replication are independent of cell type, whereas stochastic origins of replication are cell type specific (Akerman et al. 2020). We report that 74% out of the total core origins of replication have at least one G4 present in the vicinity, whereas only 24% of stochastic origins of replication had at least one G4 present, when examining G4Hunter and regex-based motifs together (Supplemental Fig. 12). By constructing a 4 kb window around the origin of replication, we mapped G4s on both the forward and reverse strands, revealing similar distributional biases as previously observed across various bacterial phyla. Consistently, G4s exhibited preferential positioning on the leading strand of the replication fork, a pattern detected using both G4Hunter and regex-based G4 identification methods (Supplemental Fig. 12). Unlike core origins of replication, stochastic origins exhibited a more pronounced signal near the replication origin, with maximum enrichment reaching 1.8-fold ∼100 bp upstream of and downstream from the origin. In contrast, core origins demonstrated a higher enrichment of 2.3-fold on both forward and reverse strands, peaking at ∼330 bp from the origin of replication (Supplemental Fig. 12). These results indicate that G4s are highly enriched at both core and stochastic origins of replication. We also investigated the G4 density across various *cis*-regulatory elements provided by SCREEN (The ENCODE Project Consortium et al. 2020) for the mouse and human genomes. Our findings suggest that loci with CTCF-bound promoter-like signatures display the highest G4 densities, for both human and the mouse (Supplemental Fig. 13), consistent with previous work (Georgakopoulos-Soares et al. 2022a).

### Experimental validation of predicted G4s

**Figure 7.**
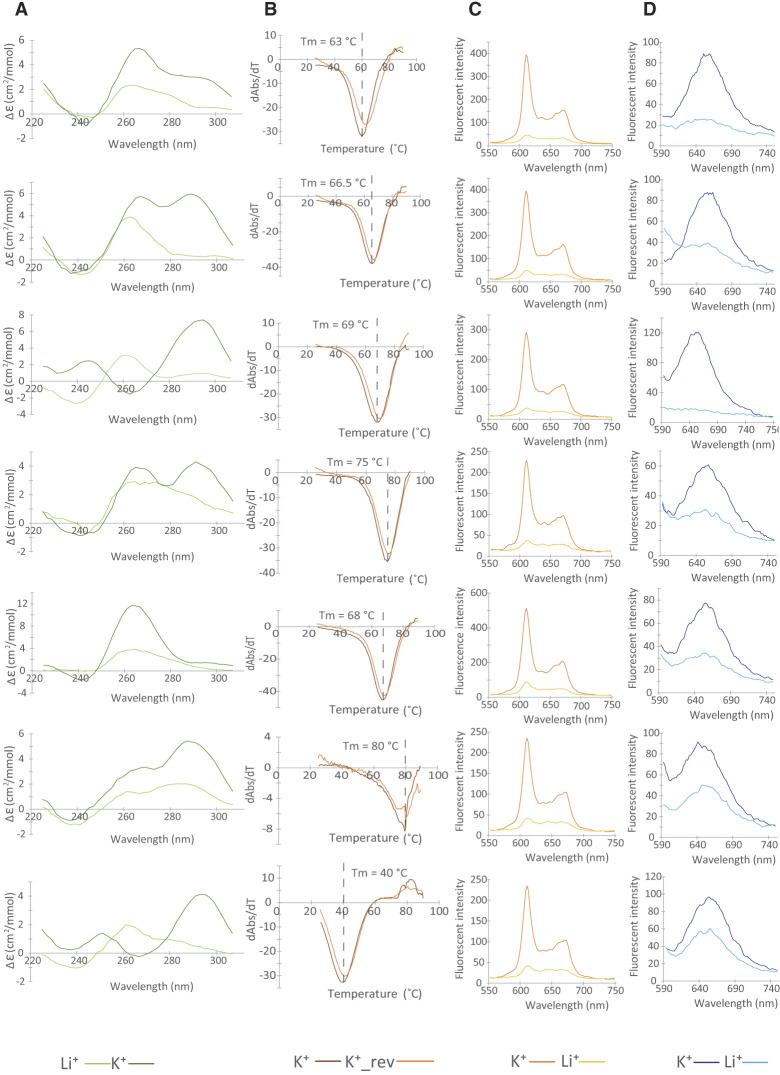
Experimentally validated G4 candidates. G4 ligand-induced G4 spectroscopic analysis reveals the G4 DNA structure formation in selected sequences from the dG4 database. (*A*) CD spectra in the presence of K^+^ and Li^+^. The overall higher signals under K^+^ conditions verify the presence of dG4 in all sequences. The positive and negative peaks at different wavelengths suggest different topologies were formed in different sequences, including parallel (negative peak at 240 nm, positive peak at 260 nm), antiparallel (negative peak at 240 nm, positive peaks at 240 nm and 295 nm), and hybrid (negative peak at 240 nm, positive peaks at 260 nm and 295 nm) topologies. (*B*) UV melting spectra in the presence of K^+^. The hypochromic shift observed at a wavelength of 295 nm is consistent with the presence of dG4 structures, with the melting temperature determined as the maximum negative value of the hypochromic shift. (*C*,*D*) NMM (*C*) and ISCH-oa1 (*D*) enhanced fluorescence spectroscopy in the presence of K^+^ and Li^+^. The fluorescence intensity is reported in units of hundreds. The higher fluorescent signals under K^+^ conditions illustrate the dG4 structure formation in all sequences.

Among the 20 most frequent G4 motifs detected from the two algorithms, we selected a subset of potential G4 DNA-forming sequences to experimentally validate their ability to adopt G4 DNA in vitro using CD spectroscopy, UV melting, and fluorescence measurements. These included G4 DNA-forming sequences that were highly prevalent across species (found in at least 2000 species) and that were only detected either by G4Hunter or the consensus regex-based G4 algorithm but not both (Supplemental Table 3). Additionally, the selected sequences were chosen to reflect G4 motifs found in the different domains of life and viruses. We conducted several spectroscopic analyses on each of the seven candidates to validate the formation of G4 structures. Initially, we utilized CD spectroscopy and UV-melting assays on DNA oligonucleotides that contained the potential G4 DNA-forming sequences. These tests were done in the presence of either lithium ions (Li^+^), which do not stabilize G4s, or potassium ions (K^+^), which do, to assess the potential and stability of G4 formation. Overall, a higher signal was found under K^+^ conditions in all CD spectra, suggesting the presence of G4 structures in all sequences tested (Fig. 7A). The CD spectra also reveal different G4 topologies formed in the different sequences, including parallel, antiparallel, and hybrid topologies (Fig. 7A). In support of the presence of G4s, our findings suggest that all candidates formed thermostable G4 structures under K^+^ conditions, which was confirmed by the hypochromic shift observed in all UV spectra, with the melting temperatures determined to be within a range of 40°C to 80°C (Fig. 7B). Additionally, we employed fluorescence-based techniques, including the use of *N*-methyl mesoporphyrin IX (NMM) ligand-enhanced fluorescence and ISCH-oa1-enhanced fluorescence experiments (Fig. 7C,D). In all cases, a higher fluorescent intensity was observed under K^+^ conditions compared to Li^+^ conditions, which indicates the formation of G4 structures in all selected sequences.

### Derivation of G4 clusters

We next performed a clustering analysis across all the identified G4s in all genomes and grouped G4 sequences based on sequence similarity. After accounting for overlapping G4 loci, the final set used for clustering comprised 118,445,719 G4s, of which 88,139,047 came from the G4Hunter-based algorithm, 8,571,114 were derived from the regex-based algorithm, and 21,735,558 were unique representatives of sequences reported by both methods. A total of 6,819,259 G4 sequence groups of one or more members were produced; 319,784 of which had 20 or more G4 sequences and were selected as the final cluster data set.

**Figure 8.**
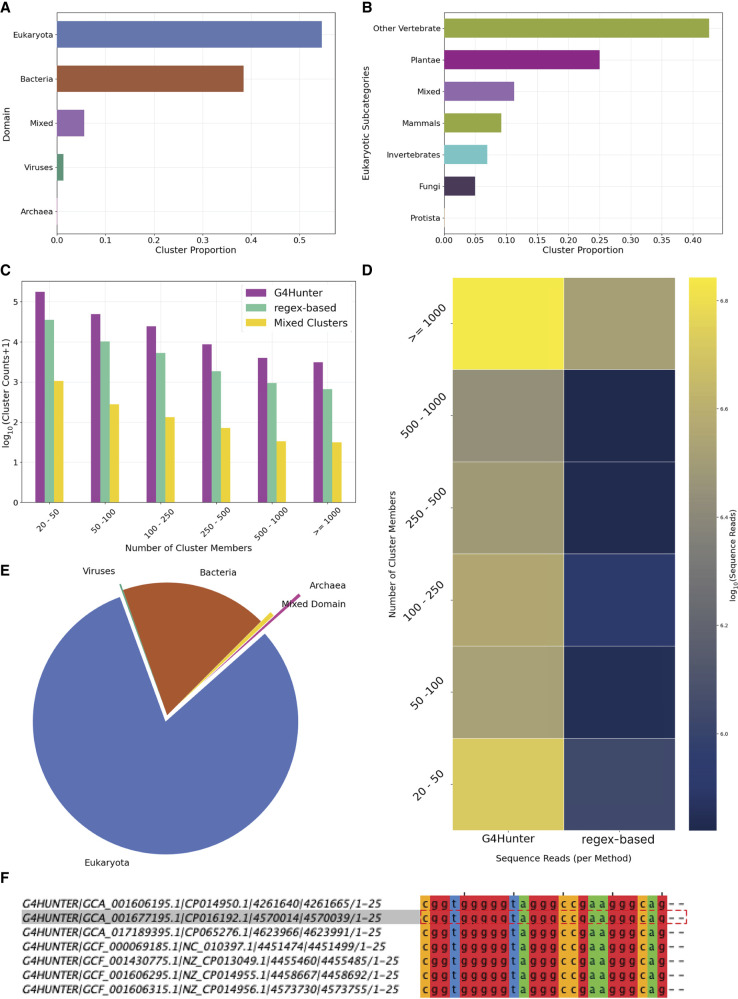
Clustering analysis of G4 sequences. (*A*) Proportion of total G4 clusters identified in each of the three domains of life and viruses, as well as those clusters that were found in at least two of these (mixed). (*B*) Proportion of clusters observed in each of the kingdoms of life. (*C*) Number of clusters based on the number of G4 sequences being members. Results are shown for the regex algorithm and the G4 Hunter algorithm. (*D*) Number of cluster members. Sequence reads were defined as the total number of G4s detected within the indicated cluster. (*E*) Origin of G4 clusters as a proportion of total sequences. (*F*) Example of G4 cluster showing the loci of the G4 sequences, and the associated sequence alignment.

We observe that the majority of G4 clusters are of eukaryotic origin followed by bacterial origin (Fig. 8A). When we further separate the G4 clusters by eukaryotic taxonomic subdivisions, we find that non-mammalian vertebrates and plants have the largest number of G4 clusters (Fig. 8B). The distribution of sequences based on their detection method shows that the majority of the clusters (263,885) contain exclusively sequences from G4Hunter (G4Hunter-based) followed by 65,868 clusters with sequences from both methods (mixed clusters) and, finally, 250 clusters exclusively containing regex-derived results (regex-based). The number of cluster members varies substantially, with the most frequent having 20–50 G4 sequence members for the G4Hunter-based, regex-based, and mixed clusters (Fig. 8C,D). Out of a total of 319,784 G4 clusters, only 135 clusters (0.04%) are mixed, meaning they are found across all four domains (bacteria, archaea, viruses, and eukaryota). The majority of the clusters are domain specific: 122,975 clusters (38.46%) are found exclusively in bacteria; 148 clusters (0.05%) are restricted to archaea; and 4339 clusters (1.36%) are unique to viruses. The largest portion, 174,435 clusters (54.56%), is found only in eukarya. Additionally, 17,880 clusters (5.59%) are composed of combinations across two or more domains but not all (Fig. 8E). Each cluster is represented by a multiple sequence alignment (MSA), which effectively models the G-quartet core of the G4 structure (example shown in Fig. 8F).

### The Quadrupia website and web interface

**Figure 9.**
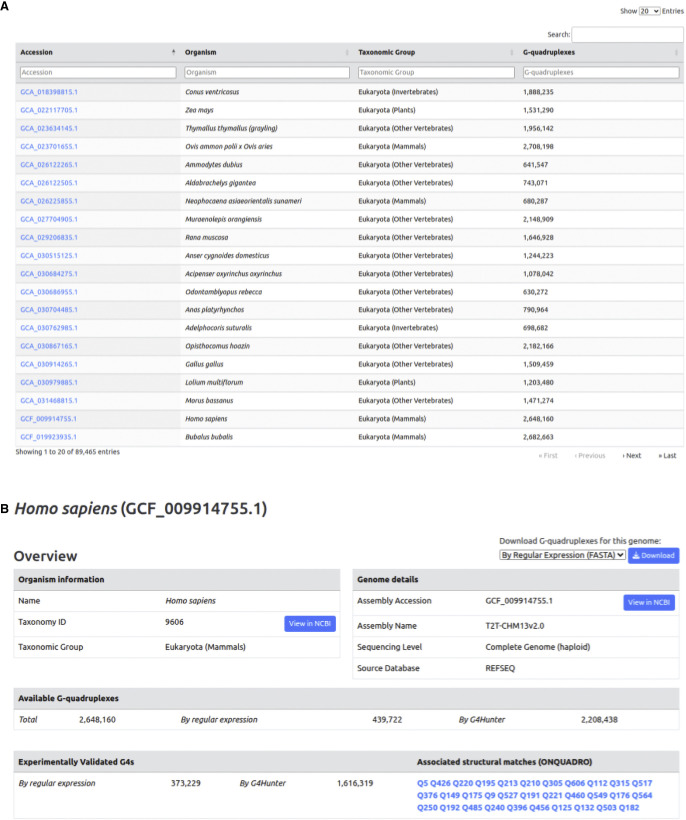

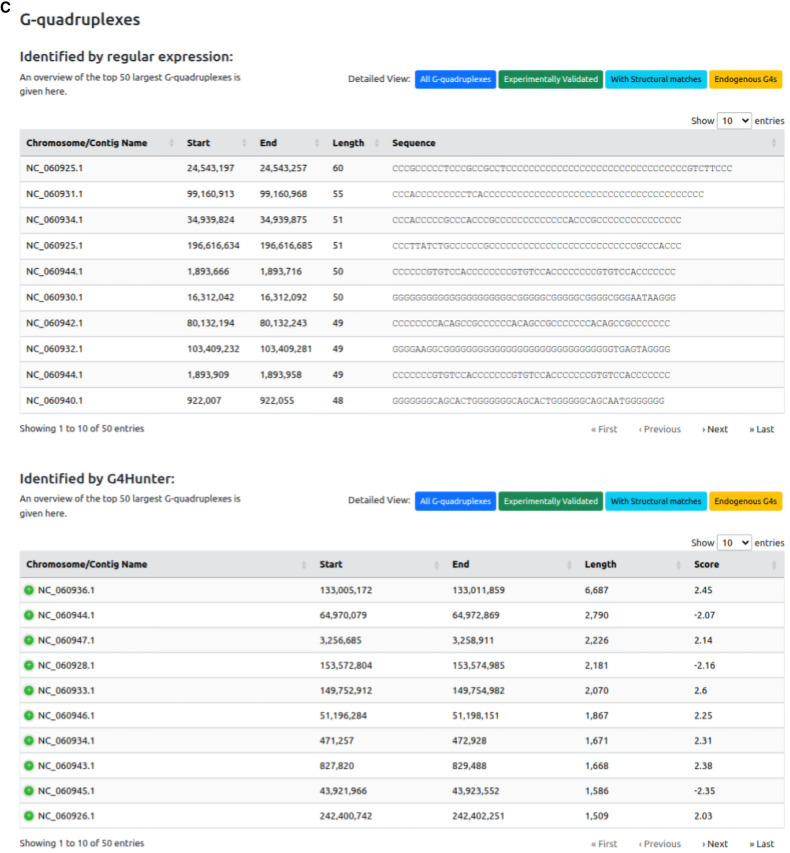
Quadrupia browse pages for reference genomes. (*A*) A table of species entries. By default, 20 entries per page are shown with the following columns: accession, organism, taxonomic group, and number of G4s. (*B*) The Quadrupia genome entry page. An example is shown for the genome of *Homo sapiens* (GCF_009914755.1). The page includes the name of the organism, the taxonomy ID, the taxonomic group, the assembly accession, the assembly name, the sequencing level, and the source database. In addition, for genomes with matches to experimentally validated G4 data, a section is displayed with the relevant information. (*C*) G4s identified using regular expressions and G4Hunter. The output is sorted by the length of the G4, and the table displays the source, chromosome/contig name, the start and end coordinates of the G4, its length, and, for G4Hunter, the G4Hunter score. The table is sortable, and the results can be downloaded. The genome page shows the top 50 largest sequences for each category, and the user has the option to explore the full set of G4s for each genome with additional annotations.

The Quadrupia website contains interactive tables and drop-down menus that enable the selection of potential G4 DNA-forming sequences based on species names or accession IDs (Fig. 9A). The data contained in Quadrupia can be accessed through the browse menu located on the Quadrupia navigation bar. A user can navigate the database for different genomes as well as taxonomic groups of bacteria, eukaryotes, archaea, and viruses. In the database, each genome is represented using its NCBI Genomes (https://www.ncbi.nlm.nih.gov/home/genomes/) accession as its primary identifier (e.g., GCF_009914755.1) The user can also specify between genomes or use a combination of both criteria. The Quadrupia browse page displays a list of genomes that can be parsed to match the selected filters (Fig. 9B). The user can further refine the search by opting to select specific species using the NCBI accession or the species name, which takes the user to the specific genome entry page (Fig. 9C). On this page, the user can view the G4s, from each of the two detection methods, associated with the selected genome. The information on this page includes the species name, the taxonomic ID, the taxonomic group, the assembly accession with an embedded link to the NCBI website, the assembly name, the sequencing level, and the source database.

Quadrupia offers experimental validation for the G4 sequences of selected genomes in three forms: (1) genomic coordinate matches to experimentally validated G4 sequences, as described in the study by Marsico et al. (2019); (2) mapping of G4 sequences to the “endogenous” G4 sequences (eG4s) of the EndoQuad database (Qian et al. 2024), a repository of manually curated, experimentally verified G4 sequences; and (3) sequence-based discovery of perfect hits to experimentally determined (X-ray crystallography and NMR) and annotated G4 structures from ONQUADRO (Zok et al. 2022). In cases of genomes with experimentally determined G4 sequences, additional information is presented, namely, the number of experimentally determined sequences and cross-references to ONQUADRO (Fig. 9C).

Finally, on the genome page, the total number of G4s identified with each of the two methods is provided as well as the top 50 longest G4s for each of the two methods. Through the genome page, users can then select and see the full list of G4 sequences with additional annotation. Annotation features for each G4 sequence include the presence of genes (CDS or nonprotein-coding) near the its coordinates, the distance of the G4 position from the TSSs and TESs, the existence of structural hits to ONQUADRO (Zok et al. 2022) or matching eG4 entries from EndoQuad (Qian et al. 2024), and, finally, the coordinate ranges that overlap with experimentally verified sequences. All G4 data are available for download in FASTA format for the sequences and as tab-delimited tables for the annotations, through the download menu at the top of the Genome page.

**Figure 10.**
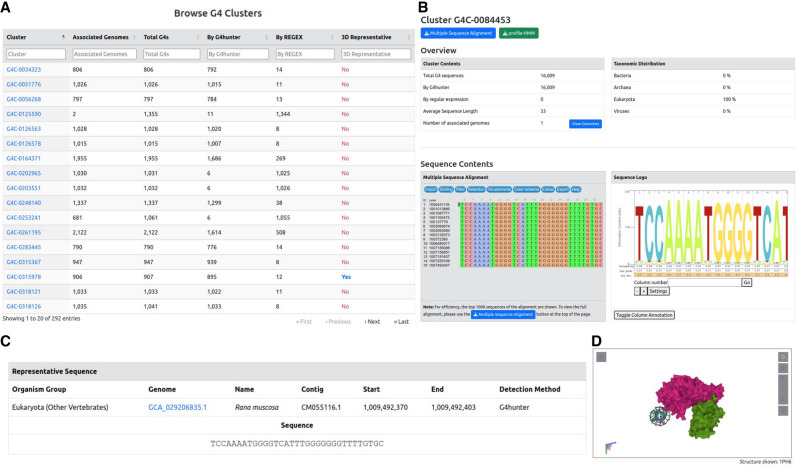
G4 cluster search and visualization. (*A*) The G4 cluster browser. Each cluster is identified by a unique identifier and is annotated by the number of associated genomes, the number of G4 sequences (total, by regular expression or G4Hunter), and, finally, the presence or absence of a 3D representative. (*B*–*D*) An example G4 cluster entry page (ID: G4C-0084453). The entry contains the basic cluster metadata, as well as the taxonomic distribution of its contents (*B*). In addition, interactive viewers are given for the cluster's MSA, HMM (in the form of a sequence logo), representative sequence (*C*), and, if available, 3D structure representative conformation (*D*).

Similar to the genomes, the database allows navigating the collection of G4 clusters (Fig. 10). Each cluster in the database is represented by a unique identifier in the form of G4-XXXXXXX (e.g., G4C-0084453) (Fig. 10A). The clusters are accessible through a separate browser, available from the top navigation menu. Each cluster can be viewed by its distinct cluster entry page and is accompanied by a number of annotation data, including taxonomic distribution, a multiple sequence alignment (MSA) and its derived hidden Markov model (HMM), and a centroid, representative sequence (Fig. 10B,C). The MSA and HMM are available for visualization in the cluster entry page through an interactive alignment browser and a sequence logo viewer, respectively. In addition, the MSA and HMM raw files are available for download through buttons at the top of the page. Finally, in cases of clusters that have sequence homology with experimentally determined G4 structures, external links to the latter are given for the PDB (Berman et al. 2000) and ONQUADRO (Zok et al. 2022) databases, and the top homolog structure is available for visualization through an interactive 3D structure viewer (Fig. 10D).

**Figure 11.**
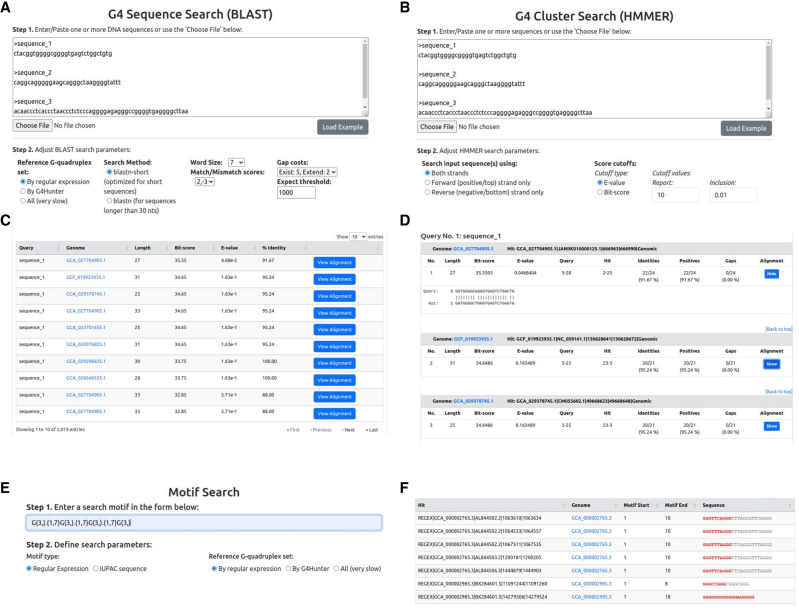
The sequence search options. (*A*) The G4 sequence search input page. Search options include defining the search algorithm (BLASTN), setting the word size, and defining the match/mismatch scores and gap penalties. (*B*) G4 cluster search. Users can choose which DNA strand to include in the search and define cutoffs for the *E*-value and bit-score metrics. (*C*,*D*) Example BLAST results. An overview (*C*) of the results presents the basic search statistics (alignment length, bit-score, *E*-value, and percentage sequence identity), and the detailed results (*D*) give the full BLAST output, including a visualization of the aligned sequences. (*E*,*F*) Input form (*E*) and example results (*F*) of the motif search functionality. Sequence motifs can be given as regular expression patterns or IUPAC sequence stretches. The results include all G4 sequences that match the submitted motif.

The database is also searchable through a dedicated advanced search page, as well as a number of sequence search options. The advanced search page allows performing complex queries against the database by combining multiple fields, such as the taxonomy, number of G4 sequences, and sequence detection method. The sequence search option, in turn, allows users to perform queries against G4 sequences or G4 clusters or to utilize sequence motifs. The G4 sequences search allows searching user-submitted DNA/RNA sequences against G4 sequences through pairwise alignments performed with BLAST (BLASTN) (Fig. 11A), whereas the G4 clusters search performs equivalent queries against Quadrupia's collection of G4 clusters, using HMM profiles and HMMER (nhmmer) (Fig. 11B). For both options, an overview of the results is given in table format (Fig. 11C), whereas individual alignments can also be accessed on demand (Fig. 11D). Complementary to the above, the motif search option allows users to submit sequence motifs as queries, either in the form of regular expressions or as IUPAC-formatted sequences (Fig. 11E). The presented results include all G4 sequences containing the submitted motif, with matching subsequences highlighted (Fig. 11F). Any regular expression motif can be used for searching in Quadrupia. Finally, Quadrupia provides a downloads page, which enables users to download the complete data sets and incorporate them in their analyses.

### Database statistics

Quadrupia offers two dedicated pages displaying statistics of its contents, accessible through the statistics option in the navigation bar. The database statistics page presents a breakdown of Quadrupia's content per organism domain, including the number of available genome assemblies, the number of G4s for each domain, and a distribution of G4 clusters with respect to their most common taxonomic group. The results are offered in the form of interactive plots. The G4 density page offers an interactive method to display the distribution of G4 motif density relative to the TSSs and TESs. Users can select and display TSS/TES plots for a specific domain (bacteria, archaea, eukaryota, or viruses), taxonomic family (e.g., Chordata), and gene type (protein-coding or nonprotein-coding).

### Documentation and help pages

The Quadrupia website has a documentation page that provides background information on G4s and a help page about how to navigate through the G4 data and the different species, as well as how to download the reference maps.

## Discussion

We have performed a systematic characterization of potential G4 DNA-forming sequences across organismal genomes spanning the tree of life, including the three domains of life and viruses, diverse kingdoms, and phyla. We introduce Quadrupia, a comprehensive database dedicated to the study and analysis of G4 DNA. Our database encompasses genome-wide maps of G4 sites for 108,534 organisms, far exceeding the number of species covered in comparison to any other G4-associated database. Computationally predicted G4 sequences can be a powerful tool in discovering new G4 sites. Sequences reported in Quadrupia have high concordance with experimentally validated sequences using G4-seq data. We integrate experimentally validated G4 structures with previous databases, such as EndoQuad (Qian et al. 2024) and ONQUADRO (Zok et al. 2022), and we also validate a subset of G4 sequences in our own experiments. The database incorporates searchable options using motif-based or BLAST-based searchers. We also perform large-scale analyses on the distribution and topology of G4s across these organismal genomes. We find that eukaryotes are the domain with the highest G4 motif density; nevertheless, there are large differences at the phylum level, with the phyla with the highest G4 motif density belonging to different domains of life and kingdoms, suggesting that G4s are genomic elements with high turnover between taxonomic subgroups.

In our analysis of G4 data across multiple species, we have incorporated species from all three domains of life (bacteria, archaea, and eukaryota) and viruses, as provided by NCBI. This broad taxonomic coverage allows for a comparative perspective on G4 distribution and conservation across evolutionary lineages. The presence of G4 motifs in such diverse organisms highlights their potential fundamental roles in genomic regulation and stability. Although the evolutionary distances among these species vary significantly, the recurrence of G4 structures in functionally important genomic sites suggests that they may have emerged owing to functional importance. This evolutionary context underscores the versatility and potential universality of G4 motifs across the tree of life. However, in our analysis, G4s were unevenly distributed across the three domains of life and viruses. Their positioning in genomic loci associated with regulatory roles, such as in the broader promoter regions, near TESs, and in intronic regions close to splice sites, highlights their involvement in mediating a spectrum of important biological functions and mechanisms, many of which may have emerged independently in different clades. We also observe that these enrichments in regulatory regions can be taxonomy specific. It is possible that G4s have dynamically evolved owing to distinct environmental influences and different organism needs.

The diversity in which G4s are distributed across different organisms, kingdoms, and phyla is suggestive of their intrinsic regulatory roles for various molecular functions and biological mechanisms. Notably, the phylum of Deinococcota displayed the highest G4 motif density using the two G4 detection algorithms. Moreover, significant enrichment of G4s was detected upstream of the TSS and downstream from the TES in the Deinococcota phylum. This is in accordance with previous work (Kota et al. 2015), in which the bacteria belonging to the Deinococcota phylum are highly resistant to environmental hazards, with high survival rates when exposed to gamma or UV radiation, and in which G4s have regulatory and radioresistant functions (Kota et al. 2015). The phylum of Peploviricota displayed a high density of G4s, appearing second in G4 motifs derived from using the regex algorithm and fourth using the G4Hunter algorithm. Viruses belonging to this phylum also constituted the majority of the top 100 viruses with the highest G4 motif densities. This viral phylum consists solely of a single order, Herpesvirales, an order of double-stranded DNA viruses, which encompasses a variety of viral species that affect animal hosts. Previous work shows that the viral cycle of the Herpes simplex virus 1 (*HSV-1*) is regulated by G4s. Because of the central role of G4s in the viral cycle, G4s have been used as drug targets for antiviral activity (Frasson et al. 2022). Additionally, we observe that viruses with eukaryotic hosts have significantly higher G4 motif densities compared with viruses with prokaryotic hosts. This might suggest that eukaryotic viruses evolved to acquire higher G4 motif densities to adapt to the G4-rich environment in their eukaryotic hosts. However, because of the large variation in G4 motif densities at the phyla level, within the same domains, further studies need to be done between viruses and their hosts at lower taxonomic levels.

Other significant G4 motif densities are found in various plant phyla such as Chlorophyta, which display a high overall G4 motif density and a significant enrichment in regulatory regions such as TSSs, TESs, and splice sites. These positional tendencies of G4s in central regulatory regions are not limited to bacteria, viruses, or plants. We highlight a significant density of G4s in various eukaryotic kingdoms such as fungi and protozoa (Fig. 2). Several fungi phyla such as Microsporidia, Basidiomycota, and Ascomycota display a high density of G4s in their genome and an enrichment of G4s within the broader promoter regions (Fig. 4). For *Aspergillus fumigatus* of the phylum Ascomycota, G4s have been associated with genes, involved with virulence, and implicated in drug resistance and could be used for the identification of novel antifungal targets (Warner et al. 2021). Similar results have been identified amongst Protozoa, with the phylum of Euglonozoa displaying similar G4 enrichment in regulatory areas.

In bacteria, we find that G4s are preferentially found in the leading strand during replication. However, we also observe that this strand asymmetry of G4s is phylum specific and is associated with the GC skew levels. There is evidence that there are more guanines than cytosines in the leading strand in bacteria (Grigoriev 1998), which could result in a higher likelihood of formation of G4s. We also observe that certain phyla, such as Cyanobacteria and Deinococcota, lack these biases and do not have strong GC skew biases. Previous research has shown that the GC skew is less pronounced in Cyanobacteria (Ohbayashi et al. 2020; Watanabe 2020), which is consistent with our findings and which could therefore account for the absence of strand asymmetry between leading and lagging strands in the distribution of G4s. G4s can be barriers to replication fork progression (Estep et al. 2019), and the observed preference of G4s for the leading orientation could influence genome stability. Future work is required to decipher the extent to which the observed differences in the frequency of G4s on the leading and lagging strands impact the mutation rate in bacterial genomes.

G4s are elements with increased mutagenicity and high turnover rate (Georgakopoulos-Soares et al. 2018, 2022d; Puig Lombardi et al. 2019; Guiblet et al. 2021a). We observe that across taxonomic groups, the G4 motif density has extreme variation, suggesting that they are plastic genomic elements driving evolution (Guiblet et al. 2021a,b). We also find that functional genomic subcompartments show large deviations in G4 motif density between taxonomic subgroups, which likely reflects the emergence of different functionalities when comparing taxonomic clades. These findings are further supported by previous results indicating that the roles of G4s in splicing have emerged during eukaryotic evolution (Georgakopoulos-Soares et al. 2022c) and by previous findings from 37 species showing that the density of G4s has increased in higher eukaryotes (Wu et al. 2021). We also note that even though this trend exists in bacteria and eukaryotes, for archaea this trend was observed only for G4 motifs derived from the regex algorithm, whereas viruses did not display this property.

To conclude, Quadrupia stands out as a multifaceted and user-friendly database, offering G4 sequences and their annotations across species and taxonomic classifications as well as several search options. Further work is required to follow individual G4s in evolutionary lineages and to understand the functional roles of conserved and recently evolved G4s. Such work can include comparative genomics and phylogenetic analyses, enabling additional insights into adaptive strategies and evolutionary constraints. Finally, previous work has indicated differences in the stability of G4s between species (Wu et al. 2021). Future studies are required to examine differences in the formation likelihood, kinetics, and stability of G4s between disparate taxonomic groups and their implications.

## Methods

### Data retrieval and parsing

Complete genomes were downloaded from the NCBI GenBank (https://www.ncbi.nlm.nih.gov/genbank/) and RefSeq databases (Benson et al. 2013; O'Leary et al. 2016). A total of 108,449 complete genomes were analyzed and integrated in the database. For each genome, the associated files for RNA and coding regions as well as the GFF gene annotation file were downloaded. Replication origins for bacteria were derived from DoriC database for complete assemblies, for circular topology, and for single origin of replication types (Dong et al. 2023).

### Identification of potential G4 DNA-forming sequences

Regular expressions (referred to as the regex algorithm) were employed to generate genome-wide G4 maps, as well as for RNA and coding regions. For each species’ genome, RNA, and coding regions, we generated the genome-wide DNA G4 maps using a regular expression of the consensus G4 motif (G ≥ 3N1–7G ≥ 3N1–7G ≥ 3N1–7G ≥ 3) (Huppert and Balasubramanian 2007). G4s were also detected using G4Hunter with parameters of a window -w = 25 and -s 1.5 (Bedrat et al. 2016; Brázda et al. 2019), with the minimum score of 1.5 being used because it has a FDR < 10% as previously estimated and recommended (Bedrat et al. 2016). Because G4s were extracted from both GenBank and RefSeq assemblies, resulting in some duplicates, unique G4s were obtained by prioritizing RefSeq assemblies over GenBank owing to a more comprehensive annotation of the RefSeq assemblies. G4s with overlapping coordinates were merged into a single sequence before further analysis was performed. Shared G4s between the G4Hunter and the regular expression methods were estimated using a minimum overlap of 50% between motifs detected from the two methods. The requirement for a minimum of 50% overlap needs to be satisfied by any one of the two sequences for them to be considered common between the two methods.

### Estimation of G4 motif density across genomes and genomic subcompartments

G4 motif density was calculated as the number of G4 base pairs over the number of base pairs examined. The average G4 motif density was calculated between organismal genomes within taxonomic groups. The G4 motif density was also examined across genomic subcompartments and was calculated as the length of overlap of G4s with each of the subcompartments divided by the total length of the subcompartments. The subcompartment coordinates were obtained from the corresponding GFF files, and overlapping annotations within a subcompartment were merged. The mean G4 motif density was calculated across species belonging to the same taxonomy either across the genome or in genomic subcompartments. Assembly accessions associated with a GFF file were included in this analysis. In all organismal genomes, we used the AGAT command line tool to annotate missing genomic subcompartments, such as missing exons in prokaryotic genomes (https://github.com/NBISweden/AGAT).

Species that did not have any relevant genomic compartment annotated in their GFF files were excluded from the analysis. The mean G4 motif density was log-transformed for the categorical plots and the heatmaps in Figure 3. To reduce noise, phyla with fewer than five species were excluded from the heatmap (Fig. 3B).

### Estimation of G4 motif density relative to TSSs, TESs, replication origins, and splice sites

To investigate the relationship between G4 sites and TSSs or TESs, we generated local windows of 500 bp around TSSs/TESs and measured the distribution of G4 base pairs across the window. To that end, we use the gene coordinates extracted from the corresponding GFF files. The enrichment was calculated as the sum of G4 occurrences at each relative position across organismal genomes over the mean of the resulting number of occurrences across the generated window. Confidence intervals were calculated as the 2.5% lowest and 97.5% highest percentile from Monte-Carlo simulations with replacement (N = 1000), in which we randomly picked an equal number of species from the domain, kingdom, or phylum that was studied.

### Origin of replication

We investigated the distribution of G4s across bacterial genomes with circular chromosomes. To that end, we downloaded the origin of replication from the single-partite data sets from the DoriC database. Each organismal genome in Doric was annotated with the start and end of origin of replication. For each available chromosome, we derived the halfway distance from the *oriC* as (start + end)/2. Afterward, we binned the chromosomal sequence in 1001 bins, with bin 500 corresponding to *oriC*. Because of the circular nature of the chromosome, we used the distance metric d(P,Q) = min(|P − Q|, ChromosomeSize − |P − Q|), in which P and Q are any two genomic positions. Consequently, the bin 1001 is identical to bin 0, whereas 500 bin is identical to the replication terminus. We associated each G4 sequence to a specific bin (in rare cases a sufficiently large G4 was assigned to more than one bin) by calculating the circular distance on the binned chromosome for that particular G4 from *oriC*. The resulting binned distribution consisted of bins containing the total number of G4s that have a distance of d units from *oriC*. Note that looking at the generated figure, a positive distance d > 0 would translate to G4 sequence being located on the left rather than on the right half-circle because *oriC* is always placed at pi/2, assuming counterclockwise orientation. Finally, we divide each position by the total mean to estimate the local enrichment of G4s. This process was repeated separately for G4s located on forward and on reverse strand, respectively, as well as for both the G4Hunter and regex methodologies. These distributions were used to generate the G4 polar scatterplots in relation to the origin of replication, using the Matplotlib polar coordinate system. Furthermore, we extracted the GC-Skew for each organismal genome. To achieve this, we split the chromosome into windows of 25bp and calculated the GC-Skew using the formula G − C/G + C. Then, following the process above, we assigned to each bin of the circular chromosome the average GC-Skew of all the windows belonging to a particular bin.

We examined the positional distribution of G4s across stochastic and core origins of replication in the human genome. We downloaded the stochastic and core origins of replication from the NCBI Gene Expression Omnibus (GEO; https://www.ncbi.nlm.nih.gov/geo/) accession number GSE128477 (Akerman et al. 2020) for the GRCh38 reference human genome, and subsequently, we performed a liftOver to the hs1 Telomere-to-Telomere human genome. We defined the replication origin as the midpoint between the start and end coordinates and extended a 4 kb window around it. We partitioned the G4s into two groups, forward and reverse, depending on the strand orientation, and subsequently, we used the BEDTools intersect command to locate the overlaps (Quinlan and Hall 2010). We counted the occurrences of G4s at a distance relative to the replication origin. Finally, to estimate the enrichment, we divided the previously estimated G4 occurrences by the window average.

### *Cis*-regulatory elements

We downloaded all the *cis*-regulatory element annotations from the SCREEN website (https://screen.encodeproject.org) by the ENCODE Project for both human (hg38) and mouse (mm10) genomes (The ENCODE Project Consortium et al. 2020). For the human genome, we performed a liftOver to map the coordinates to hs1. Subsequently, we used the BEDTools coverage command to estimate the G4 densities of *cis*-regulatory elements found in human and mouse genomes.

### Biophysical properties

We investigated the biophysical properties of G4 motifs derived from the regex algorithm and G4Hunter by decomposing each G4 into consecutive G-runs. A G-run is defined as any subsequence of G4 containing at least more than three consecutive guanines. Any G4 is composed of consecutive G-runs interrupted by a sequence that violates the pattern. We defined the loop as the union of all the nucleotide sequences between the G-runs. We used regular expressions to extract the G-runs and intervening loops for each G4 motif, and thereafter, we calculated the total frequency of each G-run length as well as the frequencies of the various loop lengths associated with each G4. Analyses were performed to compare the overall distribution across taxonomies, and at individual taxonomies.

### CD spectroscopy

Reaction samples of 2 mL containing 5 µM oligos were prepared in 10 mM LiCac (pH 7.0) and 150 mM KCl or LiCl. The samples were mixed well and denatured by heating them for 5 min at 95°C and then were cooled down to room temperature for renaturation. Data measurements were obtained using a Jasco CD J1500 spectrometer and a quartz cuvette with a path-length of 1 cm. The samples were scanned at 2 nm intervals starting from 220 nm and ending at 310 nm. The resulting data were blanked and normalized to obtain the mean residue ellipticity. The samples were scanned three times, and data were averaged. Analysis of all data was done in Spectra Manager Suite and Microsoft Excel.

### UV melting spectroscopy

Reaction samples were prepared and renatured in accordance with the CD experiment above for the KCl condition, and the UV melting assay was carried out in a Cary 3500 UV-Vis multicell Peltier spectrometer and a 1 cm path-length quartz cuvette sealed with thread seal tape. Data measurements were acquired every 0.5°C increment from 20°C to 95°C at 295 nm. All recorded data were blanked and smoothed by averaging every 10°C. Data analysis was performed in Microsoft Excel.

### G4 ligand-enhanced fluorescence spectroscopy

Reaction samples of 1 µM oligos were set up in 10 mM LiCac (pH 7.0) and 150 mM KCl or LiCl to a total volume of 100 µL and mixed well. Renaturation was conducted by heating the samples for 5 min at 95°C and were cooled down at room temperature for 15 min. After the addition of 1 µM of NMM or ISCH-OA1 ligand, the fluorescence spectra of the samples were collected using a HORIBA FluoroMax-4 fluorescence spectrophotometer and a 1 cm path-length quartz cuvette. The excitation wavelengths for NMM and ISCH-OA1 were set to 394 and 570 nm, respectively, and the spectra were collected at 550–750 nm for NMM and 590–750 nm for ISCH-OA1. Entrance slit was set to 5 nm; exit slit was set to 2 nm; and the spectra were collected every 2 nm and smoothed every three data points. All recorded data were analyzed using Microsoft Excel.

### Sequence clustering

Sequence clustering was performed for the combined data sets of the regex- and G4Hunter-derived G4 sequences. Any coordinate overlaps between the two data sets were detected as described above, and for each set of overlapping sequences, the longest was chosen as the representative candidate. Clustering was performed using the Linclust algorithm (Steinegger and Söding 2018) implemented in MMseqs2 (Steinegger and Söding 2017). Linclust was chosen as it is a *k*-mer-based method capable of clustering hundreds of millions of sequences in linear time regardless of *k*-mer length and thus is the most efficient solution for G4s. Clustering was carried out with an 80% sequence identity cutoff and a 90% bidirectional alignment coverage threshold, ensuring that sequences are sufficiently similar to be grouped together. A minimum cluster size of 20 members was set, meaning that only groups with at least 20 sequences were considered as clusters. These specific cutoff values were chosen to maintain the conservative characteristics of G4 motifs, which tend to be structurally and functionally conserved. At the same time, they aim to strike a balance by optimizing the number of sequences grouped into meaningful clusters, while reducing the number of sequences left ungrouped (singletons). This approach improves the representation of biologically relevant clusters without sacrificing the sensitivity required to detect subtle variations between motifs. Each G4 cluster was used to produce a MSA with MAFFT (Katoh et al. 2002), applying the directional adjustment parameter to consider reverse complementarity and ensure proper bidirectional alignment. The centroid sequences of each cluster, as generated by MMseqs2, were used to guide MSA generation and were defined as the representative sequences of the clusters. The MSAs were used to generate profile hidden Markov models (pHMMs) with HMMER v. 3.3.2 (Eddy 2011).

### Identification of G4 cluster structural representatives

A data set of experimentally determined G4 3D structures was constructed by parsing the records of the ONQUADRO database (retrieved May 6, 2024) (Zok et al. 2022) and matching them to their corresponding PDB (Berman et al. 2000) entries. The DNA sequences of this data set were searched against the representative sequences of the G4 clusters using BLAST+ (Camacho et al. 2009). Sequence hits were identified using a sequence identity cutoff of 80% and an alignment coverage threshold of 90% with respect to the shortest sequence. The structure with the top sequence hit to a cluster (largest sequence identity, followed by largest alignment coverage) was chosen as the cluster's structural representative.

### Mapping to experimental data sets

For the purposes of the Quadrupia database, mapping to experimentally determined G4s was performed using three data sources: (1) the experimentally determined G4 sequences, determined in the study by Marsico et al. (2019); (2) the endogenous G4 sequences provided by the EndoQuad database (Qian et al. 2024); and (3) the experimentally determined G4 3D structures hosted by ONQUADRO. Mapping of the Marsico et al. (2019) and EndoQuad G4 sequences to Quadrupia was performed using the *intersect* function of BEDTools (Quinlan and Hall 2010), with a minimum coordinate overlap of 10%, as defined previously. G4-seq data derived using PDS and K^+^ conditions were concatenated and compared against computationally predicted G4s. For the ONQUADRO structures, matches were identified by performing BLAST searches, with a 100% sequence identity threshold and a 100% alignment coverage with respect to the sequence of the ONQUADRO structures to identify G4 sequences that contain all features required to form a stable G4 3D structure.

### Database implementation

The front end of Quadrupia was implemented in HTML, CSS, and JavaScript. The back end is supported by the Apache web server and the Slim Framework v. 4.0, with server-side operations handled by PHP and, when required, Python. The genome metadata is stored in a MySQL relational database. The Quadrupia website layout is designed using the Bootstrap v. 5 framework, jQuery, and the DataTables library. Data visualization is performed using MSAviewer (Yachdav et al. 2016) for MSAs, SkyLign (Wheeler et al. 2014) for sequence logos, and Molstar (Sehnal et al. 2021) for 3D structures. Sequence queries are performed using BLAST+ (Camacho et al. 2009) for sequences and *nhmmscan* (Wheeler and Eddy 2013) for pHMMs. An additional option to perform motif-based queries is also offered, implemented using Python.

### Software availability

The code for Quadrupia is available at GitHub (https://github.com/Georgakopoulos-Soares-lab/Quadrupia) and as Supplemental Code.

## Supplemental Material

Supplement 1

Supplement 2

## References

[GR279790CHAC1] Adamczyk B, Zurkowski M, Szachniuk M, Zok T. 2023. WebTetrado: a webserver to explore quadruplexes in nucleic acid 3D structures. Nucleic Acids Res 51: W607–W612. 10.1093/nar/gkad346 37158242 PMC10320137

[GR279790CHAC2] Akerman I, Kasaai B, Bazarova A, Sang PB, Peiffer I, Artufel M, Derelle R, Smith G, Rodriguez-Martinez M, Romano M, 2020. A predictable conserved DNA base composition signature defines human core DNA replication origins. Nat Commun 11: 4826. 10.1038/s41467-020-18527-0 32958757 PMC7506530

[GR279790CHAC3] Bartas M, Čutová M, Brázda V, Kaura P, Št′astný J, Kolomazník J, Coufal J, Goswami P, Červeň J, Pečinka P. 2019. The presence and localization of G-quadruplex forming sequences in the domain of bacteria. Molecules 24: 1711. 10.3390/molecules24091711 31052562 PMC6539912

[GR279790CHAC4] Bedrat A, Lacroix L, Mergny J-L. 2016. Re-evaluation of G-quadruplex propensity with G4Hunter. Nucleic Acids Res 44: 1746–1759. 10.1093/nar/gkw006 26792894 PMC4770238

[GR279790CHAC5] Belmonte-Reche E, Morales JC. 2020. G4-iM grinder: when size and frequency matter. G-quadruplex, i-Motif and higher order structure search and analysis tool. NAR Genom Bioinform 2: lqz005. 10.1093/nargab/lqz005 33575559 PMC7671307

[GR279790CHAC6] Benson DA, Cavanaugh M, Clark K, Karsch-Mizrachi I, Lipman DJ, Ostell J, Sayers EW. 2013. Genbank. Nucleic Acids Res 41: D36–D42. 10.1093/nar/gks1195 23193287 PMC3531190

[GR279790CHAC7] Berman HM, Westbrook J, Feng Z, Gilliland G, Bhat TN, Weissig H, Shindyalov IN, Bourne PE. 2000. The protein data bank. Nucleic Acids Res 28: 235–242. 10.1093/nar/28.1.235 10592235 PMC102472

[GR279790CHAC8] Besnard E, Babled A, Lapasset L, Milhavet O, Parrinello H, Dantec C, Marin J-M, Lemaitre J-M. 2012. Unraveling cell type–specific and reprogrammable human replication origin signatures associated with G-quadruplex consensus motifs. Nat Struct Mol Biol 19: 837–844. 10.1038/nsmb.2339 22751019

[GR279790CHAC01] Biffi G, Tannahill D, McCafferty J, Balasubramanian S. 2013. Quantitative visualization of DNA G-quadruplex structures in human cells. Nat Chem 5: 182–186. 10.1038/nchem.1548 23422559 PMC3622242

[GR279790CHAC9] Brázda V, Kolomazník J, Lýsek J, Bartas M, Fojta M, Št′astný J, Mergny J-L. 2019. G4Hunter web application: a web server for G-quadruplex prediction. Bioinformatics 35: 3493–3495. 10.1093/bioinformatics/btz087 30721922 PMC6748775

[GR279790CHAC10] Brázda V, Luo Y, Bartas M, Kaura P, Porubiaková O, Št′astný J, Pečinka P, Verga D, Da Cunha V, Takahashi TS, 2020. G-Quadruplexes in the Archaea domain. Biomolecules 10: 1349. 10.3390/biom10091349 32967357 PMC7565180

[GR279790CHAC11] Brooks TA, Kendrick S, Hurley L. 2010. Making sense of G-quadruplex and i-motif functions in oncogene promoters. FEBS J 277: 3459–3469. 10.1111/j.1742-4658.2010.07759.x 20670278 PMC2971675

[GR279790CHAC12] Bugaut A, Balasubramanian S. 2012. 5′-UTR RNA G-quadruplexes: translation regulation and targeting. Nucleic Acids Res 40: 4727–4741. 10.1093/nar/gks068 22351747 PMC3367173

[GR279790CHAC13] Camacho C, Coulouris G, Avagyan V, Ma N, Papadopoulos J, Bealer K, Madden TL. 2009. BLAST+: architecture and applications. BMC Bioinformatics 10: 421. 10.1186/1471-2105-10-421 20003500 PMC2803857

[GR279790CHAC02] Cer RZ, Donohue DE, Mudunuri US, Temiz NA, Loss MA, Starner NJ, Halusa GN, Volfovsky N, Yi M, Luke BT, 2013. Non-B DB v2.0: a database of predicted non-B DNA-forming motifs and its associated tools. Nucleic Acids Res 41(Database issue): D94–D100. 10.1093/nar/gks955 23125372 PMC3531222

[GR279790CHAC14] Chambers VS, Marsico G, Boutell JM, Di Antonio M, Smith GP, Balasubramanian S. 2015. High-throughput sequencing of DNA G-quadruplex structures in the human genome. Nat Biotechnol 33: 877–881. 10.1038/nbt.3295 26192317

[GR279790CHAC15] Chan C-Y, Umar MI, Kwok CK. 2019. Spectroscopic analysis reveals the effect of a single nucleotide bulge on G-quadruplex structures. Chem Commun 55: 2616–2619. 10.1039/C8CC09929D 30724299

[GR279790CHAC16] Chen MC, Tippana R, Demeshkina NA, Murat P, Balasubramanian S, Myong S, Ferré-D'Amaré AR. 2018. Structural basis of G-quadruplex unfolding by the DEAH/RHA helicase DHX36. Nature 558: 465–469. 10.1038/s41586-018-0209-9 29899445 PMC6261253

[GR279790CHAC17] Del Villar-Guerra R, Trent JO, Chaires JB. 2018. G-quadruplex secondary structure obtained from circular dichroism spectroscopy. Angew Chem Int Ed Engl 57: 7171–7175. 10.1002/anie.201709184 29076232 PMC5920796

[GR279790CHAC18] Dhapola P, Chowdhury S. 2016. QuadBase2: web server for multiplexed guanine quadruplex mining and visualization. Nucleic Acids Res 44: W277–W283. 10.1093/nar/gkw425 27185890 PMC4987949

[GR279790CHAC19] Di Antonio M, Ponjavic A, Radzevičius A, Ranasinghe RT, Catalano M, Zhang X, Shen J, Needham L-M, Lee SF, Klenerman D, 2020. Single-molecule visualization of DNA G-quadruplex formation in live cells. Nat Chem 12: 832–837. 10.1038/s41557-020-0506-4 32690897 PMC7610488

[GR279790CHAC20] Di Salvo M, Pinatel E, Talà A, Fondi M, Peano C, Alifano P. 2018. G4PromFinder: an algorithm for predicting transcription promoters in GC-rich bacterial genomes based on AT-rich elements and G-quadruplex motifs. BMC Bioinformatics 19: 36. 10.1186/s12859-018-2049-x 29409441 PMC5801747

[GR279790CHAC21] Dong M-J, Luo H, Gao F. 2023. Doric 12.0: an updated database of replication origins in both complete and draft prokaryotic genomes. Nucleic Acids Res 51: D117–D120. 10.1093/nar/gkac964 36305822 PMC9825612

[GR279790CHAC22] Eddy SR. 2011. Accelerated profile HMM searches. PLoS Comput Biol 7: e1002195. 10.1371/journal.pcbi.1002195 22039361 PMC3197634

[GR279790CHAC23] Eddy J, Maizels N. 2006. Gene function correlates with potential for G4 DNA formation in the human genome. Nucleic Acids Res 34: 3887–3896. 10.1093/nar/gkl529 16914419 PMC1557811

[GR279790CHAC24] The ENCODE Project Consortium, Moore JE, Purcaro MJ, Pratt HE, Epstein CB, Shoresh N, Adrian J, Kawli T, Davis CA, Dobin A, 2020. Expanded encyclopaedias of DNA elements in the human and mouse genomes. Nature 583: 699–710. 10.1038/s41586-020-2493-4 32728249 PMC7410828

[GR279790CHAC25] Estep KN, Butler TJ, Ding J, Brosh RM. 2019. G4-interacting DNA helicases and polymerases: potential therapeutic targets. Curr Med Chem 26: 2881–2897. 10.2174/0929867324666171116123345 29149833 PMC6663639

[GR279790CHAC26] Frasson I, Soldà P, Nadai M, Tassinari M, Scalabrin M, Gokhale V, Hurley LH, Richter SN. 2022. Quindoline-derivatives display potent G-quadruplex-mediated antiviral activity against herpes simplex virus 1. Antiviral Res 208: 105432. 10.1016/j.antiviral.2022.105432 36228762 PMC9720158

[GR279790CHAC27] Garant J-M, Perreault J-P, Scott MS. 2017. Motif independent identification of potential RNA G-quadruplexes by G4RNA screener. Bioinformatics 33: 3532–3537. 10.1093/bioinformatics/btx498 29036425 PMC5870565

[GR279790CHAC28] Ge F, Wang Y, Li H, Zhang R, Wang X, Li Q, Liang Z, Yang L. 2019. Plant-GQ: an integrative database of G-quadruplex in plant. J Comput Biol 26: 1013–1019. 10.1089/cmb.2019.0010 30958698

[GR279790CHAC29] Georgakopoulos-Soares I, Morganella S, Jain N, Hemberg M, Nik-Zainal S. 2018. Noncanonical secondary structures arising from non-B DNA motifs are determinants of mutagenesis. Genome Res 28: 1264–1271. 10.1101/gr.231688.117 30104284 PMC6120622

[GR279790CHAC30] Georgakopoulos-Soares I, Chan CSY, Ahituv N, Hemberg M. 2022a. High-throughput techniques enable advances in the roles of DNA and RNA secondary structures in transcriptional and post-transcriptional gene regulation. Genome Biol 23: 159. 10.1186/s13059-022-02727-6 35851062 PMC9290270

[GR279790CHAC31] Georgakopoulos-Soares I, Parada GE, Hemberg M. 2022b. Secondary structures in RNA synthesis, splicing and translation. Comput Struct Biotechnol J 20: 2871–2884. 10.1016/j.csbj.2022.05.041 35765654 PMC9198270

[GR279790CHAC32] Georgakopoulos-Soares I, Parada GE, Wong HY, Medhi R, Furlan G, Munita R, Miska EA, Kwok CK, Hemberg M. 2022c. Alternative splicing modulation by G-quadruplexes. Nat Commun 13: 2404. 10.1038/s41467-022-30071-7 35504902 PMC9065059

[GR279790CHAC33] Georgakopoulos-Soares I, Victorino J, Parada GE, Agarwal V, Zhao J, Wong HY, Umar MI, Elor O, Muhwezi A, An J-Y, 2022d. High-throughput characterization of the role of non-B DNA motifs on promoter function. Cell Genom 2: 100111. 10.1016/j.xgen.2022.100111 35573091 PMC9105345

[GR279790CHAC34] Ghosh A, Largy E, Gabelica V. 2021. DNA G-quadruplexes for native mass spectrometry in potassium: a database of validated structures in electrospray-compatible conditions. Nucleic Acids Res 49: 2333–2345. 10.1093/nar/gkab039 33555347 PMC7913678

[GR279790CHAC35] Ghosh A, Pandey SP, Joshi DC, Rana P, Ansari AH, Sundar JS, Singh P, Khan Y, Ekka MK, Chakraborty D, 2023. Identification of G-quadruplex structures in MALAT1 lncRNA that interact with nucleolin and nucleophosmin. Nucleic Acids Res 51: 9415–9431. 10.1093/nar/gkad639 37558241 PMC11314421

[GR279790CHAC36] Grigoriev A. 1998. Analyzing genomes with cumulative skew diagrams. Nucleic Acids Res 26: 2286–2290. 10.1093/nar/26.10.2286 9580676 PMC147580

[GR279790CHAC37] Guiblet WM, Cremona MA, Harris RS, Chen D, Eckert KA, Chiaromonte F, Huang Y-F, Makova KD. 2021a. Non-B DNA: a major contributor to small- and large-scale variation in nucleotide substitution frequencies across the genome. Nucleic Acids Res 49: 1497–1516. 10.1093/nar/gkaa1269 33450015 PMC7897504

[GR279790CHAC38] Guiblet WM, DeGiorgio M, Cheng X, Chiaromonte F, Eckert KA, Huang Y-F, Makova KD. 2021b. Selection and thermostability suggest G-quadruplexes are novel functional elements of the human genome. Genome Res 31: 1136–1149. 10.1101/gr.269589.120 34187812 PMC8256861

[GR279790CHAC39] Hänsel-Hertsch R, Beraldi D, Lensing SV, Marsico G, Zyner K, Parry A, Di Antonio M, Pike J, Kimura H, Narita M, 2016. G-quadruplex structures mark human regulatory chromatin. Nat Genet 48: 1267–1272. 10.1038/ng.3662 27618450

[GR279790CHAC40] Harkness RW V, Mittermaier AK. 2017. G-quadruplex dynamics. Biochim Biophys Acta Proteins Proteomics 1865: 1544–1554. 10.1016/j.bbapap.2017.06.012 28642152

[GR279790CHAC41] Huang H, Zhang J, Harvey SE, Hu X, Cheng C. 2017. RNA G-quadruplex secondary structure promotes alternative splicing via the RNA-binding protein hnRNPF. Genes Dev 31: 2296–2309. 10.1101/gad.305862.117 29269483 PMC5769772

[GR279790CHAC42] Huppert JL, Balasubramanian S. 2005. Prevalence of quadruplexes in the human genome. Nucleic Acids Res 33: 2908–2916. 10.1093/nar/gki609 15914667 PMC1140081

[GR279790CHAC43] Huppert JL, Balasubramanian S. 2007. G-quadruplexes in promoters throughout the human genome. Nucleic Acids Res 35: 406–413. 10.1093/nar/gkl1057 17169996 PMC1802602

[GR279790CHAC44] Huppert JL, Bugaut A, Kumari S, Balasubramanian S. 2008. G-quadruplexes: the beginning and end of UTRs. Nucleic Acids Res 36: 6260–6268. 10.1093/nar/gkn511 18832370 PMC2577360

[GR279790CHAC45] Ida R, Wu G. 2008. Direct NMR detection of alkali metal ions bound to G-quadruplex DNA. J Am Chem Soc 130: 3590–3602. 10.1021/ja709975z 18293981

[GR279790CHAC46] Katoh K, Misawa K, Kuma K, Miyata T. 2002. MAFFT: a novel method for rapid multiple sequence alignment based on fast Fourier transform. Nucleic Acids Res 30: 3059–3066. 10.1093/nar/gkf436 12136088 PMC135756

[GR279790CHAC47] Kikin O, D'Antonio L, Bagga PS. 2006. QGRS mapper: a web-based server for predicting G-quadruplexes in nucleotide sequences. Nucleic Acids Res 34: W676–W682. 10.1093/nar/gkl253 16845096 PMC1538864

[GR279790CHAC48] Kikin O, Zappala Z, D'Antonio L, Bagga PS. 2008. GRSDB2 and GRS_UTRdb: databases of quadruplex forming G-rich sequences in pre-mRNAs and mRNAs. Nucleic Acids Res 36: D141–D148. 10.1093/nar/gkm982 18045785 PMC2238929

[GR279790CHAC49] Kota S, Dhamodharan V, Pradeepkumar PI, Misra HS. 2015. G-quadruplex forming structural motifs in the genome of *Deinococcus radiodurans* and their regulatory roles in promoter functions. Appl Microbiol Biotechnol 99: 9761–9769. 10.1007/s00253-015-6808-6 26201493

[GR279790CHAC50] Kumari S, Bugaut A, Huppert JL, Balasubramanian S. 2007. An RNA G-quadruplex in the 5′ UTR of the NRAS proto-oncogene modulates translation. Nat Chem Biol 3: 218–221. 10.1038/nchembio864 17322877 PMC2206252

[GR279790CHAC51] Kwok CK, Merrick CJ. 2017. G-quadruplexes: prediction, characterization, and biological application. Trends Biotechnol 35: 997–1013. 10.1016/j.tibtech.2017.06.012 28755976

[GR279790CHAC52] Kwok CK, Marsico G, Sahakyan AB, Chambers VS, Balasubramanian S. 2016. rG4-seq reveals widespread formation of G-quadruplex structures in the human transcriptome. Nat Methods 13: 841–844. 10.1038/nmeth.3965 27571552

[GR279790CHAC53] Lago S, Nadai M, Cernilogar FM, Kazerani M, Domíniguez Moreno H, Schotta G, Richter SN. 2021. Promoter G-quadruplexes and transcription factors cooperate to shape the cell type-specific transcriptome. Nat Commun 12: 3885. 10.1038/s41467-021-24198-2 34162892 PMC8222265

[GR279790CHAC54] Lavezzo E, Berselli M, Frasson I, Perrone R, Palù G, Brazzale AR, Richter SN, Toppo S. 2018. G-quadruplex forming sequences in the genome of all known human viruses: a comprehensive guide. PLoS Comput Biol 14: e1006675. 10.1371/journal.pcbi.1006675 30543627 PMC6307822

[GR279790CHAC55] Lee DSM, Ghanem LR, Barash Y. 2020. Integrative analysis reveals RNA G-quadruplexes in UTRs are selectively constrained and enriched for functional associations. Nat Commun 11: 527. 10.1038/s41467-020-14404-y 31988292 PMC6985247

[GR279790CHAC56] Li Q, Xiang J-F, Yang Q-F, Sun H-X, Guan A-J, Tang Y-L. 2013. G4LDB: a database for discovering and studying G-quadruplex ligands. Nucleic Acids Res 41: D1115–D1123. 10.1093/nar/gks1101 23161677 PMC3531060

[GR279790CHAC57] Li L, Williams P, Ren W, Wang MY, Gao Z, Miao W, Huang M, Song J, Wang Y. 2021. YY1 interacts with guanine quadruplexes to regulate DNA looping and gene expression. Nat Chem Biol 17: 161–168. 10.1038/s41589-020-00695-1 33199912 PMC7854983

[GR279790CHAC58] Li Z, Qian SH, Wang F, Mohamed HI, Yang G, Chen Z-X, Wei D. 2022. G-quadruplexes in genomes of viruses infecting eukaryotes or prokaryotes are under different selection pressures from hosts. J Genet Genomics 49: 20–29. 10.1016/j.jgg.2021.08.018 34601118

[GR279790CHAC59] Li G, Su G, Wang Y, Wang W, Shi J, Li D, Sui G. 2023. Integrative genomic analyses of promoter G-quadruplexes reveal their selective constraint and association with gene activation. Commun Biol 6: 625. 10.1038/s42003-023-05015-6 37301913 PMC10257653

[GR279790CHAC60] Lombardi EP, Londoño-Vallejo A. 2020. A guide to computational methods for G-quadruplex prediction. Nucleic Acids Res 48: 1603. 10.1093/nar/gkaa033 31943112 PMC7026631

[GR279790CHAC61] Lu XJ. 2020. DSSR-enabled innovative schematics of 3D nucleic acid structures with PyMOL. Nucleic Acids Res 48: e74. 10.1093/nar/gkaa426 32442277 PMC7367123

[GR279790CHAC62] Lu X-J, Bussemaker HJ, Olson WK. 2015. DSSR: an integrated software tool for dissecting the spatial structure of RNA. Nucleic Acids Res 43: e142. 10.1093/nar/gkv716 26184874 PMC4666379

[GR279790CHAC63] Lyu K, Chow EY-C, Mou X, Chan T-F, Kwok CK. 2021. RNA G-quadruplexes (rG4s): genomics and biological functions. Nucleic Acids Res 49: 5426–5450. 10.1093/nar/gkab187 33772593 PMC8191793

[GR279790CHAC64] Makova KD, Weissensteiner MH. 2023. Noncanonical DNA structures are drivers of genome evolution. Trends Genet 39: 109–124. 10.1016/j.tig.2022.11.005 36604282 PMC9877202

[GR279790CHAC65] Marsico G, Chambers VS, Sahakyan AB, McCauley P, Boutell JM, Antonio MD, Balasubramanian S. 2019. Whole genome experimental maps of DNA G-quadruplexes in multiple species. Nucleic Acids Res 47: 3862–3874. 10.1093/nar/gkz179 30892612 PMC6486626

[GR279790CHAC66] Mergny J-L, Lacroix L. 2009. UV melting of G-quadruplexes. Curr Protoc Nucleic Acid Chem 37: 17.1.1–17.1.15. 10.1002/0471142700.nc1701s37 19488970

[GR279790CHAC67] Merrikh CN, Merrikh H. 2018. Gene inversion potentiates bacterial evolvability and virulence. Nat Commun 9: 4662. 10.1038/s41467-018-07110-3 30405125 PMC6220195

[GR279790CHAC68] Métifiot M, Amrane S, Litvak S, Andreola M-L. 2014. G-quadruplexes in viruses: function and potential therapeutic applications. Nucleic Acids Res 42: 12352–12366. 10.1093/nar/gku999 25332402 PMC4227801

[GR279790CHAC69] Mishra SK, Tawani A, Mishra A, Kumar A. 2016. G4IPDB: a database for G-quadruplex structure forming nucleic acid interacting proteins. Sci Rep 6: 38144. 10.1038/srep38144 27905517 PMC5131279

[GR279790CHAC70] Miskiewicz J, Sarzynska J, Szachniuk M. 2021. How bioinformatics resources work with G4 RNAs. Brief Bioinform 22: bbaa201. 10.1093/bib/bbaa201 32898859 PMC8138894

[GR279790CHAC71] Murat P, Marsico G, Herdy B, Ghanbarian AT, Portella G, Balasubramanian S. 2018. RNA G-quadruplexes at upstream open reading frames cause DHX36- and DHX9-dependent translation of human mRNAs. Genome Biol 19: 229. 10.1186/s13059-018-1602-2 30591072 PMC6307142

[GR279790CHAC72] Ohbayashi R, Hirooka S, Onuma R, Kanesaki Y, Hirose Y, Kobayashi Y, Fujiwara T, Furusawa C, Miyagishima S-Y. 2020. Evolutionary changes in DnaA-dependent chromosomal replication in cyanobacteria. Front Microbiol 11: 786. 10.3389/fmicb.2020.00786 32411117 PMC7198777

[GR279790CHAC73] O'Leary NA, Wright MW, Brister JR, Ciufo S, Haddad D, McVeigh R, Rajput B, Robbertse B, Smith-White B, Ako-Adjei D, 2016. Reference sequence (RefSeq) database at NCBI: current status, taxonomic expansion, and functional annotation. Nucleic Acids Res 44: D733–D745. 10.1093/nar/gkv1189 26553804 PMC4702849

[GR279790CHAC74] Parkinson GN, Lee MPH, Neidle S. 2002. Crystal structure of parallel quadruplexes from human telomeric DNA. Nature 417: 876–880. 10.1038/nature755 12050675

[GR279790CHAC75] Puig Lombardi E, Holmes A, Verga D, Teulade-Fichou M-P, Nicolas A, Londoño-Vallejo A. 2019. Thermodynamically stable and genetically unstable G-quadruplexes are depleted in genomes across species. Nucleic Acids Res 47: 6098–6113. 10.1093/nar/gkz463 31114920 PMC6614823

[GR279790CHAC77] Qian SH, Shi M-W, Xiong Y-L, Zhang Y, Zhang Z-H, Song X-M, Deng X-Y, Chen Z-X. 2024. EndoQuad: a comprehensive genome-wide experimentally validated endogenous G-quadruplex database. Nucleic Acids Res 52: D72–D80. 10.1093/nar/gkad966 37904589 PMC10767823

[GR279790CHAC78] Qiu Z, Yu S, Zheng L, Lou Y, Chen X, Xuan F. 2025. Global burden of thyroid cancer in adolescents and young adults (aged 15–39 years) from 1990 to 2021: a systematic analysis of the Global Burden of Disease Study 2021. PLoS One 20: e0318605. 10.1371/journal.pone.0318605 39951481 PMC11828416

[GR279790CHAC79] Quinlan AR, Hall IM. 2010. BEDTools: a flexible suite of utilities for comparing genomic features. Bioinformatics 26: 841–842. 10.1093/bioinformatics/btq033 20110278 PMC2832824

[GR279790CHAC80] Rawal P, Kummarasetti VBR, Ravindran J, Kumar N, Halder K, Sharma R, Mukerji M, Das SK, Chowdhury S. 2006. Genome-wide prediction of G4 DNA as regulatory motifs: role in *Escherichia coli* global regulation. Genome Res 16: 644–655. 10.1101/gr.4508806 16651665 PMC1457047

[GR279790CHAC81] Robinson J, Raguseo F, Nuccio SP, Liano D, Di Antonio M. 2021. DNA G-quadruplex structures: more than simple roadblocks to transcription? Nucleic Acids Res 49: 8419–8431. 10.1093/nar/gkab609 34255847 PMC8421137

[GR279790CHAC82] Sahakyan AB, Chambers VS, Marsico G, Santner T, Di Antonio M, Balasubramanian S. 2017. Machine learning model for sequence-driven DNA G-quadruplex formation. Sci Rep 7: 14535. 10.1038/s41598-017-14017-4 29109402 PMC5673958

[GR279790CHAC83] Saranathan N, Vivekanandan P. 2019. G-quadruplexes: more than just a kink in microbial genomes. Trends Microbiol 27: 148–163. 10.1016/j.tim.2018.08.011 30224157 PMC7127049

[GR279790CHAC84] Sehnal D, Bittrich S, Deshpande M, Svobodová R, Berka K, Bazgier V, Velankar S, Burley SK, Koča J, Rose AS. 2021. Mol*Viewer: modern web app for 3D visualization and analysis of large biomolecular structures. Nucleic Acids Res 49: W431–W437. 10.1093/nar/gkab314 33956157 PMC8262734

[GR279790CHAC85] Shao X, Zhang W, Umar MI, Wong HY, Seng Z, Xie Y, Zhang Y, Yang L, Kwok CK, Deng X. 2020. RNA G-Quadruplex structures mediate gene regulation in bacteria. mBio 11: e02926-19. 10.1128/mBio.02926-19 31964733 PMC6974567

[GR279790CHAC86] Shen J, Varshney D, Simeone A, Zhang X, Adhikari S, Tannahill D, Balasubramanian S. 2021. Promoter G-quadruplex folding precedes transcription and is controlled by chromatin. Genome Biol 22: 143. 10.1186/s13059-021-02346-7 33962653 PMC8103603

[GR279790CHAC87] Simone R, Fratta P, Neidle S, Parkinson GN, Isaacs AM. 2015. G-quadruplexes: emerging roles in neurodegenerative diseases and the non-coding transcriptome. FEBS Lett 589: 1653–1668. 10.1016/j.febslet.2015.05.003 25979174

[GR279790CHAC88] Song J, Perreault J-P, Topisirovic I, Richard S. 2016. RNA G-quadruplexes and their potential regulatory roles in translation. Translation (Austin) 4: e1244031. 10.1080/21690731.2016.1244031 28090421 PMC5173311

[GR279790CHAC89] Spiegel J, Adhikari S, Balasubramanian S. 2020. The structure and function of DNA G-quadruplexes. Trends Chem 2: 123–136. 10.1016/j.trechm.2019.07.002 32923997 PMC7472594

[GR279790CHAC90] Spiegel J, Cuesta SM, Adhikari S, Hänsel-Hertsch R, Tannahill D, Balasubramanian S. 2021. G-quadruplexes are transcription factor binding hubs in human chromatin. Genome Biol 22: 117. 10.1186/s13059-021-02324-z 33892767 PMC8063395

[GR279790CHAC91] Steinegger M, Söding J. 2017. MMseqs2 enables sensitive protein sequence searching for the analysis of massive data sets. Nat Biotechnol 35: 1026–1028. 10.1038/nbt.3988 29035372

[GR279790CHAC92] Steinegger M, Söding J. 2018. Clustering huge protein sequence sets in linear time. Nat Commun 9: 2542. 10.1038/s41467-018-04964-5 29959318 PMC6026198

[GR279790CHAC93] Summers PA, Lewis BW, Gonzalez-Garcia J, Porreca RM, Lim AHM, Cadinu P, Martin-Pintado N, Mann DJ, Edel JB, Vannier JB, 2021. Visualising G-quadruplex DNA dynamics in live cells by fluorescence lifetime imaging microscopy. Nat Commun 12: 162. 10.1038/s41467-020-20414-7 33420085 PMC7794231

[GR279790CHAC94] Vannutelli A, Schell LLN, Perreault J-P, Ouangraoua A. 2023. GAIA: G-quadruplexes in alive creature database. Nucleic Acids Res 51: D135–D140. 10.1093/nar/gkac657 35971612 PMC9825426

[GR279790CHAC95] Wang G, Vasquez KM. 2023. Dynamic alternative DNA structures in biology and disease. Nat Rev Genet 24: 211–234. 10.1038/s41576-022-00539-9 36316397 PMC11634456

[GR279790CHAC96] Wang E, Thombre R, Shah Y, Latanich R, Wang J. 2021. G-Quadruplexes as pathogenic drivers in neurodegenerative disorders. Nucleic Acids Res 49: 4816–4830. 10.1093/nar/gkab164 33784396 PMC8136783

[GR279790CHAC97] Wang Y-H, Yang Q-F, Lin X, Chen D, Wang Z-Y, Chen B, Han H-Y, Chen H-D, Cai K-C, Li Q, 2022. G4LDB 2.2: a database for discovering and studying G-quadruplex and i-Motif ligands. Nucleic Acids Res 50: D150–D160. 10.1093/nar/gkab952 34718746 PMC8728129

[GR279790CHAC98] Warner EF, Bohálová N, Brázda V, Waller ZAE, Bidula S. 2021. Analysis of putative quadruplex-forming sequences in fungal genomes: novel antifungal targets? Microbial Genomics 7: 000570. 10.1099/mgen.0.000570 33956596 PMC8209732

[GR279790CHAC99] Watanabe S. 2020. Cyanobacterial multi-copy chromosomes and their replication. Biosci Biotechnol Biochem 84: 1309–1321. 10.1080/09168451.2020.1736983 32157949

[GR279790CHAC100] Wheeler TJ, Eddy SR. 2013. nhmmer: DNA homology search with profile HMMs. Bioinformatics 29: 2487–2489. 10.1093/bioinformatics/btt403 23842809 PMC3777106

[GR279790CHAC101] Wheeler TJ, Clements J, Finn RD. 2014. Skylign: a tool for creating informative, interactive logos representing sequence alignments and profile hidden Markov models. BMC Bioinformatics 15: 7. 10.1186/1471-2105-15-7 24410852 PMC3893531

[GR279790CHAC04] Wong HM, Stegle O, Rodgers S, Huppert JL. 2010. A toolbox for predicting G-quadruplex formation and stability. J Nucleic Acids 2010: 564946. 10.4061/2010/564946 20725630 PMC2915886

[GR279790CHAC102] Wu F, Niu K, Cui Y, Li C, Lyu M, Ren Y, Chen Y, Deng H, Huang L, Zheng S, 2021. Genome-wide analysis of DNA G-quadruplex motifs across 37 species provides insights into G4 evolution. Commun Biol 4: 98. 10.1038/s42003-020-01643-4 33483610 PMC7822830

[GR279790CHAC103] Wulfridge P, Yan Q, Rell N, Doherty J, Jacobson S, Offley S, Deliard S, Feng K, Phillips-Cremins JE, Gardini A, 2023. G-quadruplexes associated with R-loops promote CTCF binding. Mol Cell 83: 3064–3079.e5. 10.1016/j.molcel.2023.07.009 37552993 PMC10529333

[GR279790CHAC104] Yachdav G, Wilzbach S, Rauscher B, Sheridan R, Sillitoe I, Procter J, Lewis SE, Rost B, Goldberg T. 2016. MSAViewer: interactive JavaScript visualization of multiple sequence alignments. Bioinformatics 32: 3501–3503. 10.1093/bioinformatics/btw474 27412096 PMC5181560

[GR279790CHAC105] Yadav VK, Abraham JK, Mani P, Kulshrestha R, Chowdhury S. 2007. QuadBase: genome-wide database of G4 DNA—occurrence and conservation in human, chimpanzee, mouse and rat promoters and 146 microbes. Nucleic Acids Res 36: D381–D385. 10.1093/nar/gkm781 17962308 PMC2238983

[GR279790CHAC106] Yu H, Qi Y, Yang B, Yang X, Ding Y. 2023. G4Atlas: a comprehensive transcriptome-wide G-quadruplex database. Nucleic Acids Res 51: D126–D134. 10.1093/nar/gkac896 36243987 PMC9825586

[GR279790CHAC107] Yuan J, He X, Wang Y. 2023. G-quadruplex DNA contributes to RNA polymerase II-mediated 3D chromatin architecture. Nucleic Acids Res 51: 8434–8446. 10.1093/nar/gkad588 37427784 PMC10484665

[GR279790CHAC108] Zhang R, Lin Y, Zhang C-T. 2008. Greglist: a database listing potential G-quadruplex regulated genes. Nucleic Acids Res 36: D372–D376. 10.1093/nar/gkm787 17916572 PMC2238908

[GR279790CHAC109] Zhang R, Shu H, Wang Y, Tao T, Tu J, Wang C, Mergny J-L, Sun X. 2023. G-Quadruplex structures are key modulators of somatic structural variants in cancers. Cancer Res 83: 1234–1248. 10.1158/0008-5472.CAN-22-3089 36791413 PMC10102852

[GR279790CHAC110] Zhao J, Chow EY-C, Yeung PY, Zhang QC, Chan T-F, Kwok CK. 2022. Enhanced transcriptome-wide RNA G-quadruplex sequencing for low RNA input samples with rG4-seq 2.0. BMC Biol 20: 257. 10.1186/s12915-022-01448-3 36372875 PMC9661767

[GR279790CHAC111] Zhong H-S, Dong M-J, Gao F. 2023. G4Bank: a database of experimentally identified DNA G-quadruplex sequences. Interdiscip Sci 15: 515–523. 10.1007/s12539-023-00577-9 37389723

[GR279790CHAC112] Zok T, Popenda M, Szachniuk M. 2020. ElTetrado: a tool for identification and classification of tetrads and quadruplexes. BMC Bioinformatics 21: 40. 10.1186/s12859-020-3385-1 32005130 PMC6995151

[GR279790CHAC113] Zok T, Kraszewska N, Miskiewicz J, Pielacinska P, Zurkowski M, Szachniuk M. 2022. ONQUADRO: a database of experimentally determined quadruplex structures. Nucleic Acids Res 50: D253–D258. 10.1093/nar/gkab1118 34986600 PMC8728301

