# Supplementary material for: Quadrupia provides a comprehensive catalog of G-quadruplexes across genomes from the tree of life
Source: Genome Res. 2025 Nov;35(11):2578–600. doi: 10.1101/gr.279790.124 (PMC12581993; doi:10.1101/gr.279790.124)
Supplement: Supplement 1 [file Supplemental_Material.pdf]

## Supplemental Figures

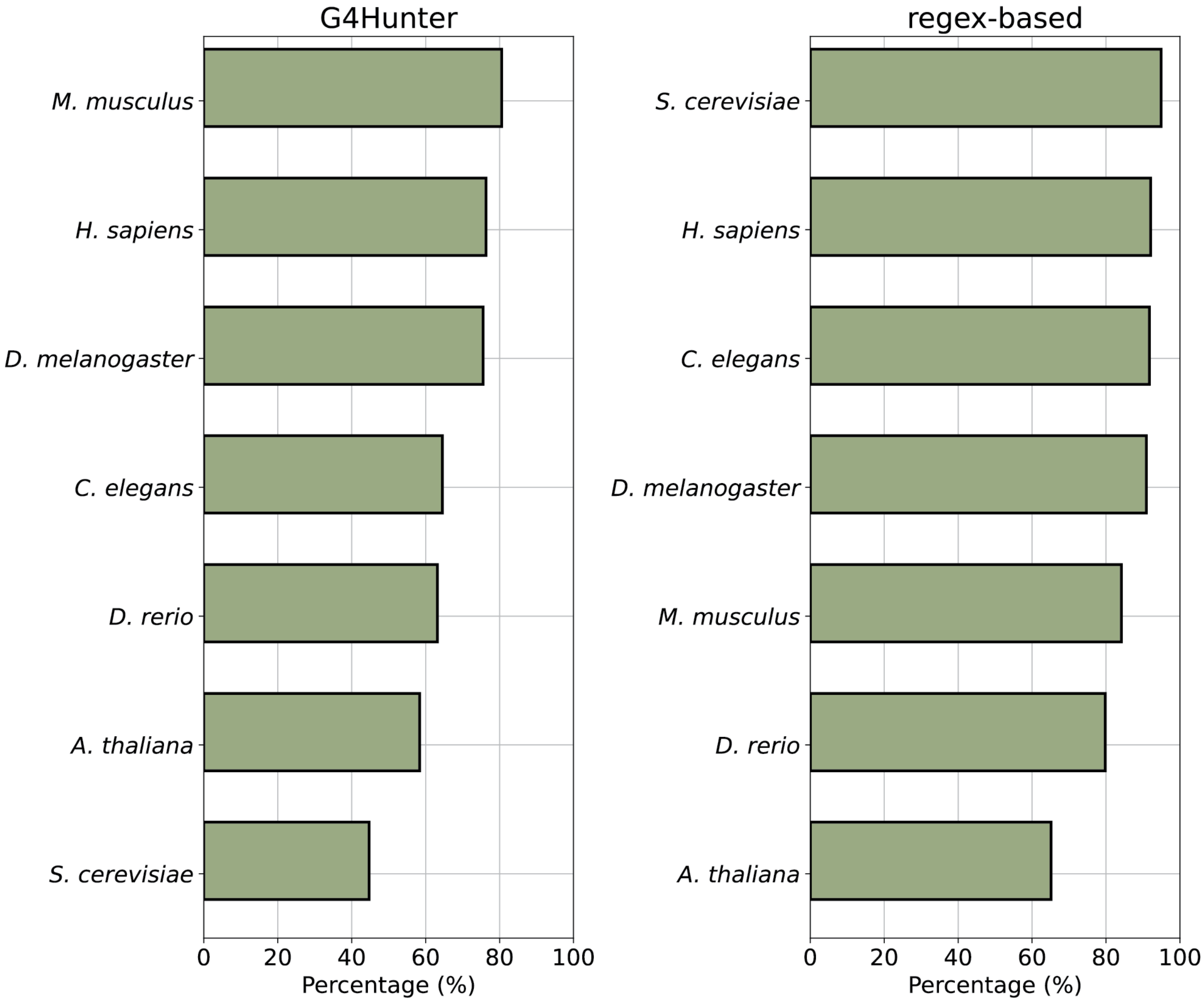


**Supplemental Figure 1: Examination of percent overlap between G4s identified with G4Hunter and regex-based approaches and G4-seq data.**

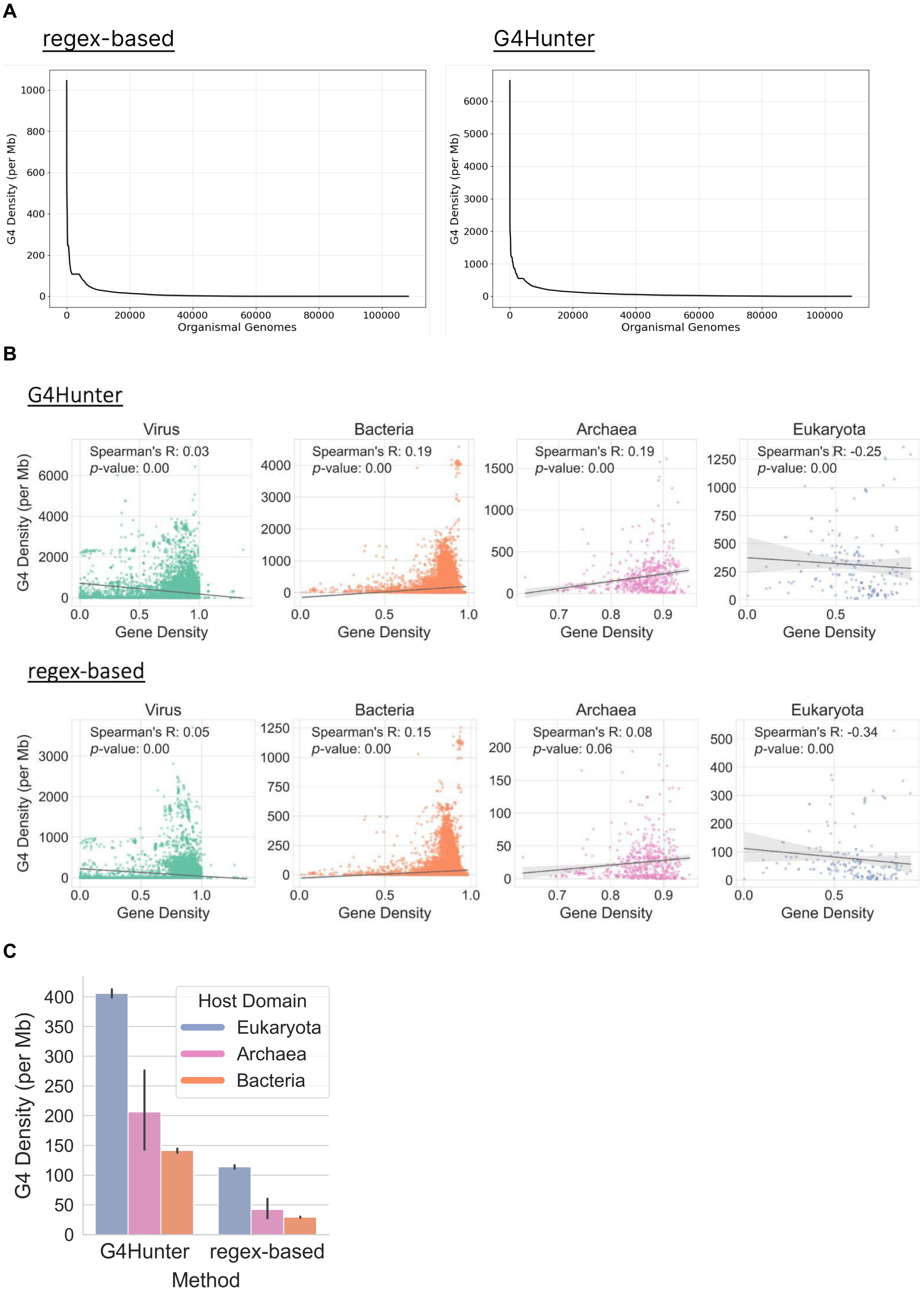


**Supplemental Figure 2: Distribution of potential G4 DNA-forming sequences across organismal genomes and taxa. A)** G4 motif density per Mb per organismal genome. **B)**

Association between the gene density of a genome and the G4 motif density, stratified by taxonomic subdivision in the three domains of life and viruses. **C)** G4 densities of viral genomes stratified by the domain of their hosts.

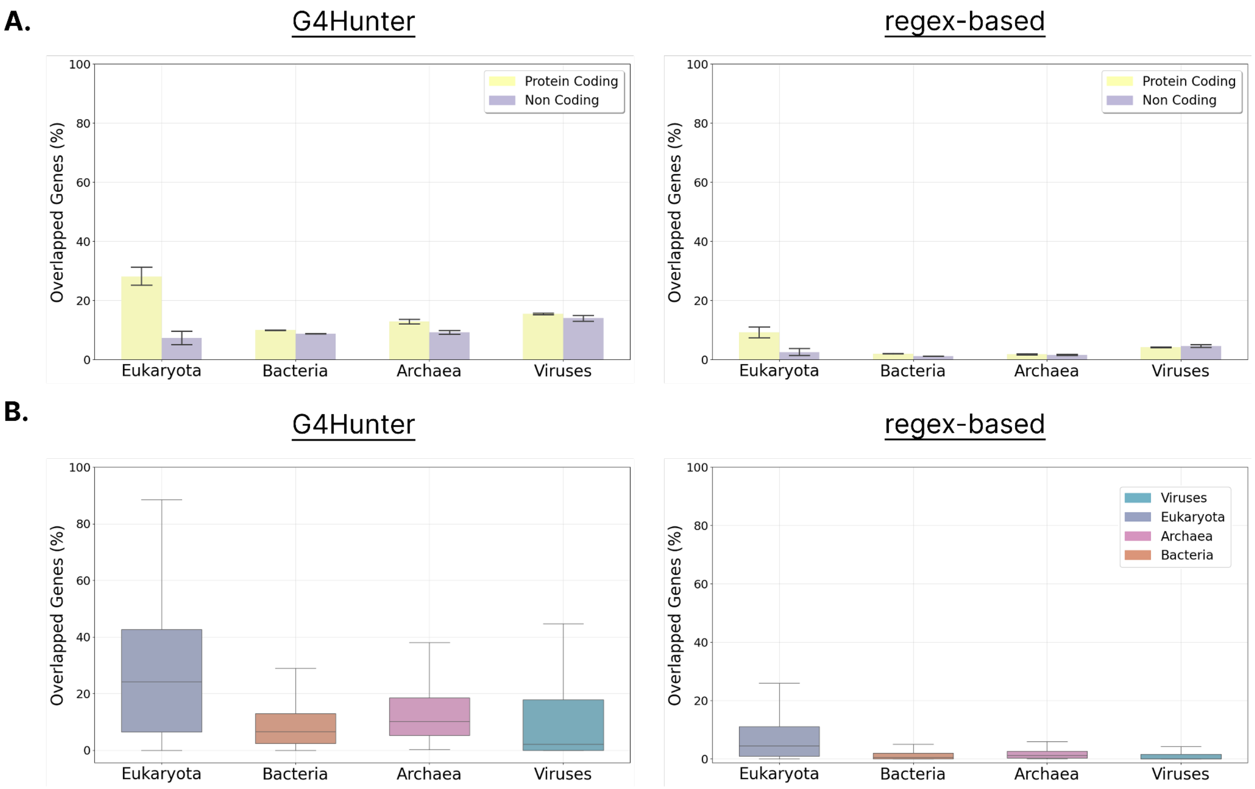


**Supplemental Figure 3: Proportion of G4s overlapping genes.**

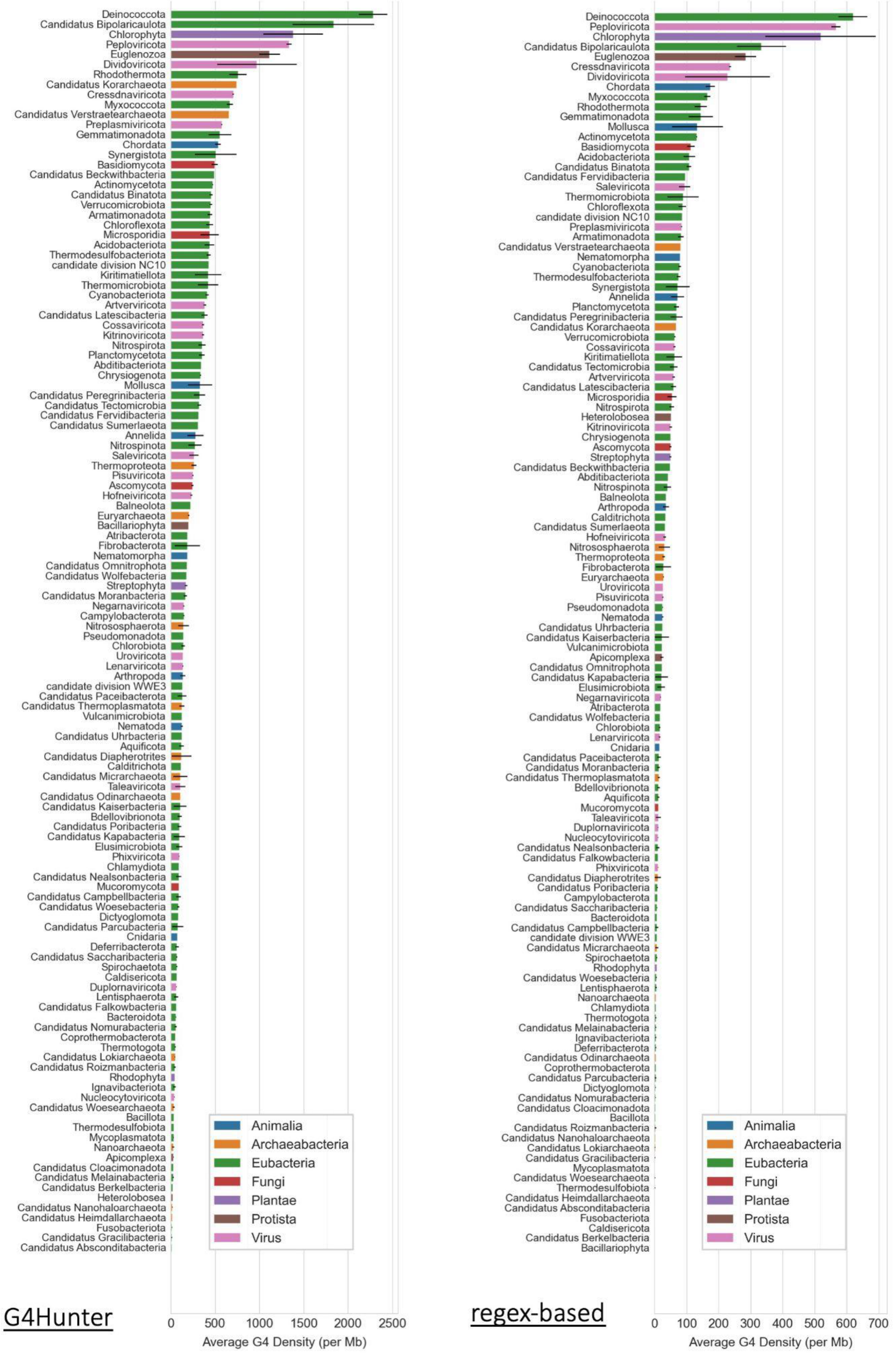


**Supplemental Figure 4: Density of G4s in each phylum. Error bars show standard deviation from the mean. All phyla are displayed.**

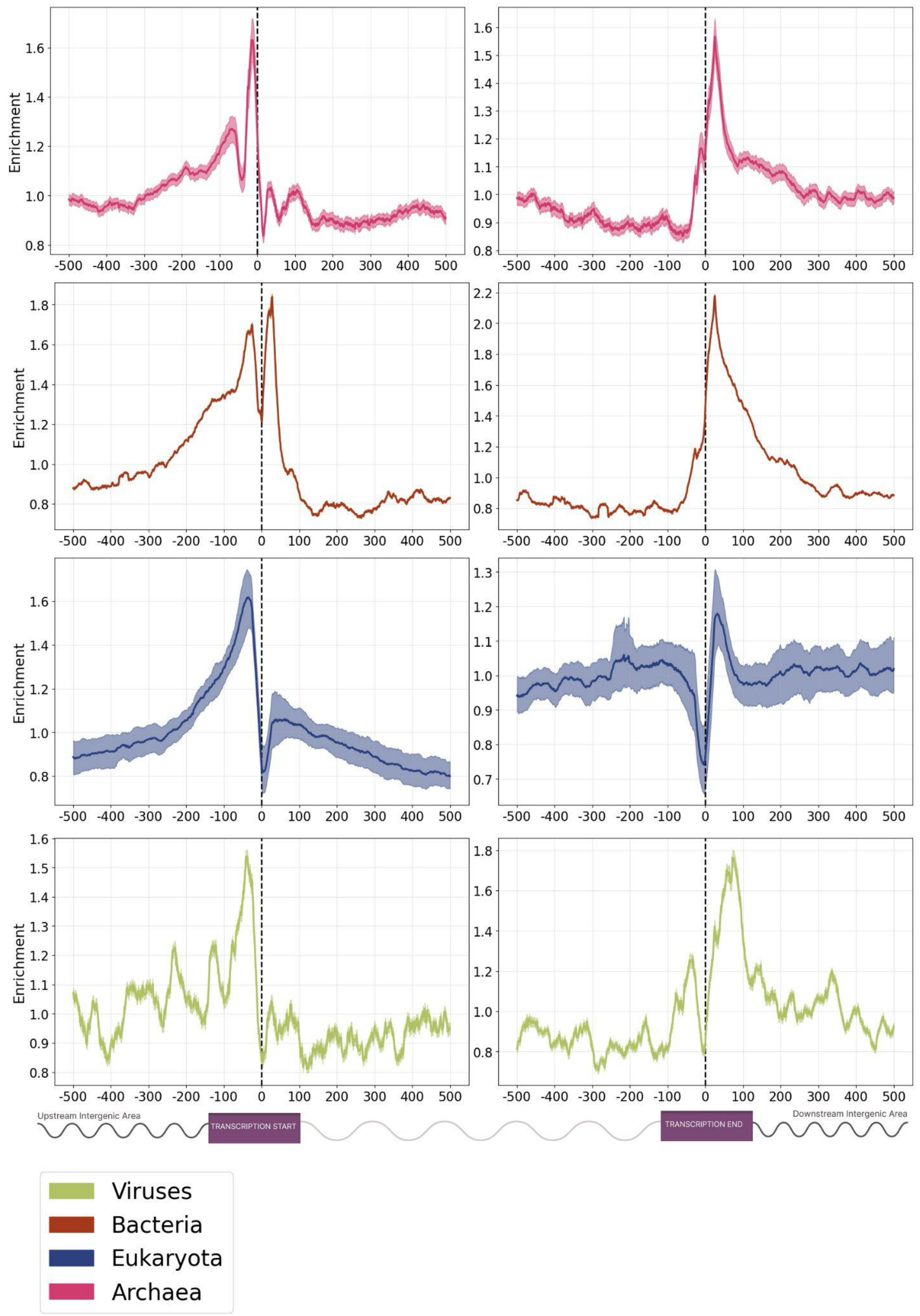
Enrichment
-500 -400 -300 -200 -100 0 100 200 300 400 500
Upstream Intergenic Area
TRANSCRIPTION START
TRANSCRIPTION END
Downstream Intergenic Area
Viruses
Bacteria
Eukaryota
Archaea

**Supplemental Figure 5: The topography of G4s relative to transcription start and transcription end sites across the tree of life.** G4 distribution across the three domains of life and viruses derived using the G4Hunter algorithm. Confidence intervals represent the 2.5% lowest and 97.5% highest percentile from Monte-Carlo simulations with replacement (N=1,000).

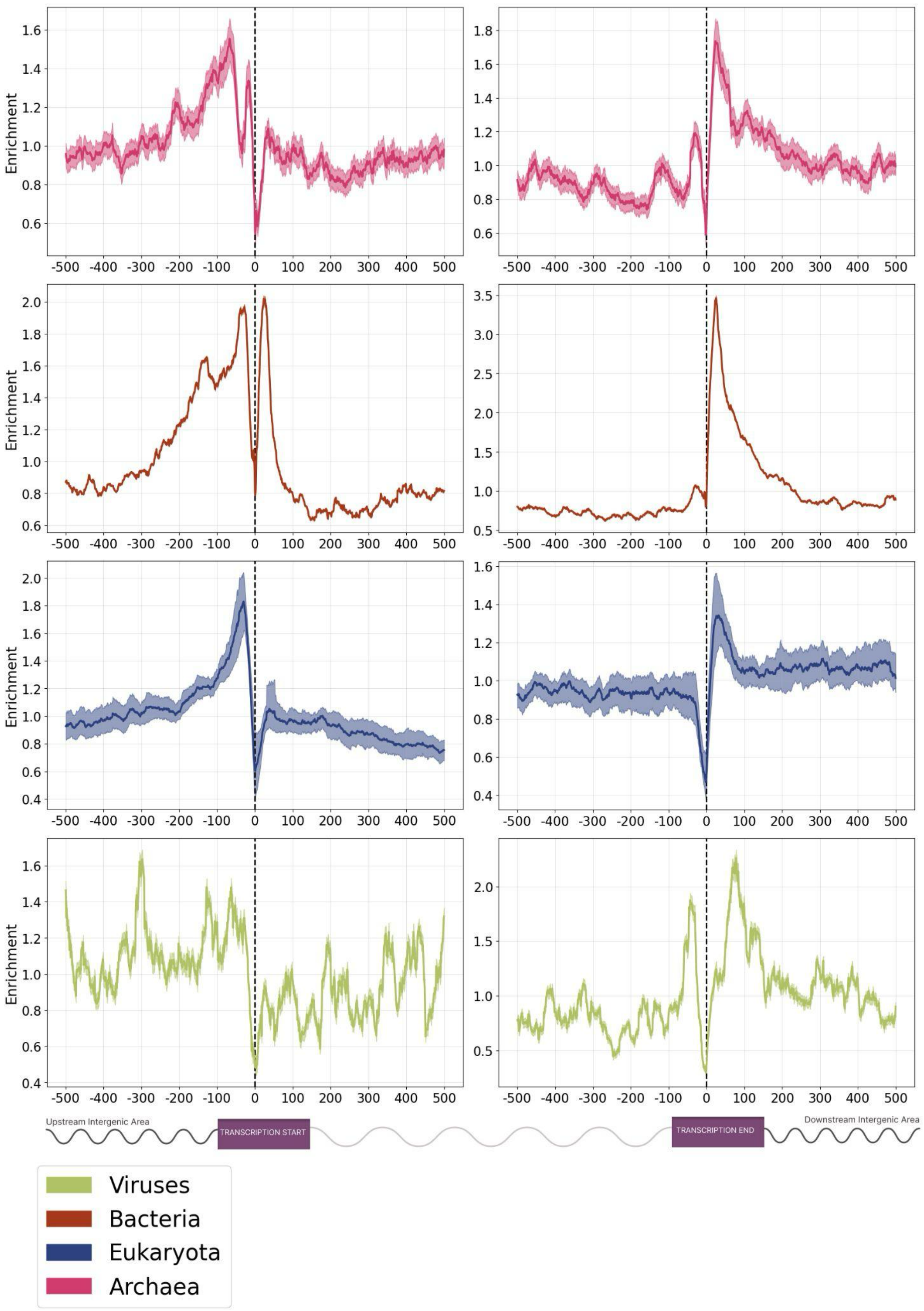

Enrichment
Upstream Intergenic Area
TRANSCRIPTION START
TRANSCRIPTION END
Downstream Intergenic Area
Viruses
Bacteria
Eukaryota
Archaea

**Supplemental Figure 6: The topography of G4s relative to transcription start and transcription end sites across the tree of life.** G4 distribution across the three domains of life and viruses derived using the regular expression-based algorithm. Confidence intervals represent the 2.5% lowest and 97.5% highest percentile from Monte-Carlo simulations with replacement (N=1,000).

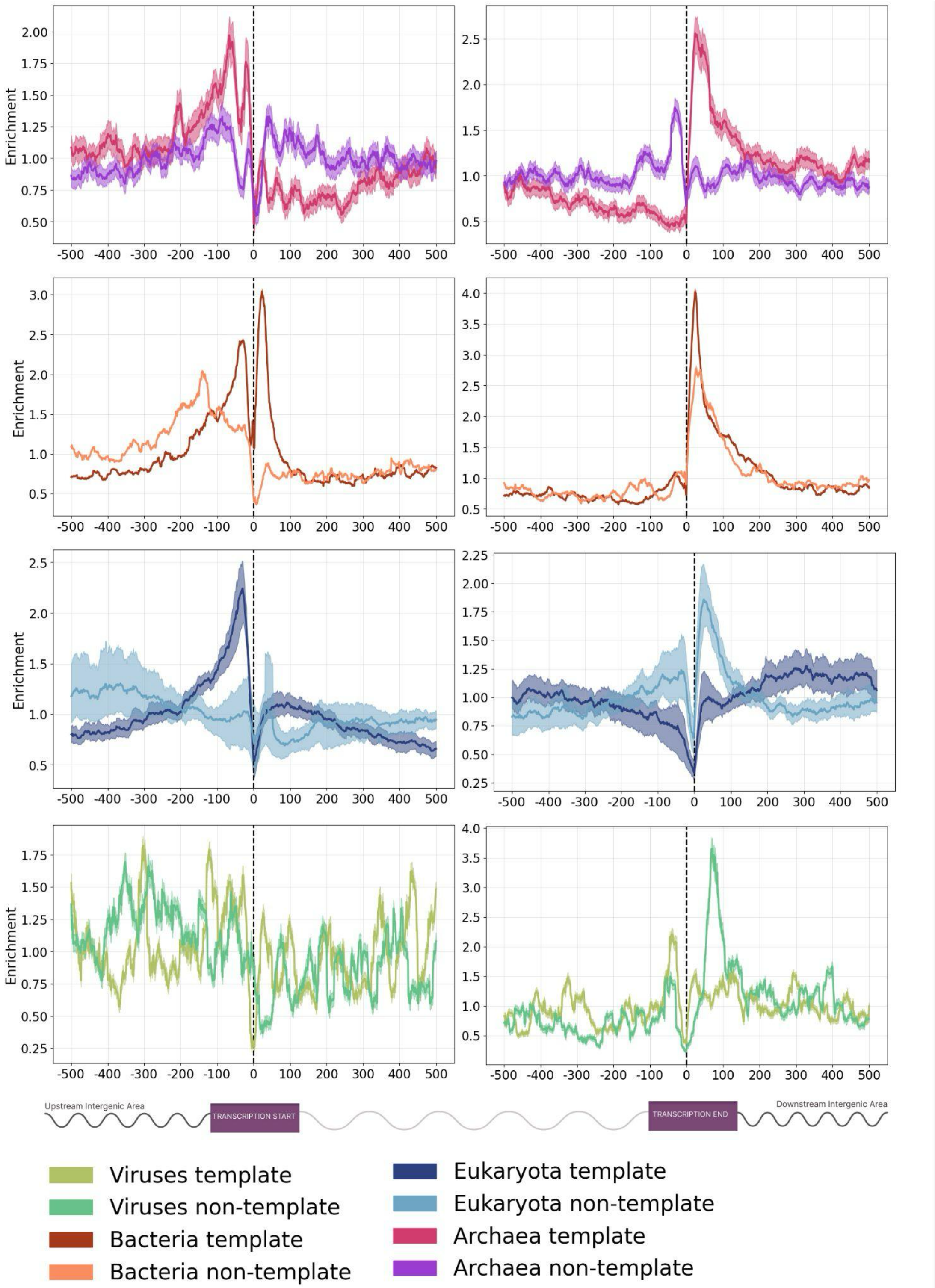


**Supplemental Figure 7: The topography of G4s relative to transcription start and transcription end sites across the tree of life at the template and non-template strands.** G4 distribution across the three domains of life and viruses derived using the regular expression-based algorithm. Confidence intervals represent the 2.5% lowest and 97.5% highest percentile from Monte-Carlo simulations with replacement (N=1,000).

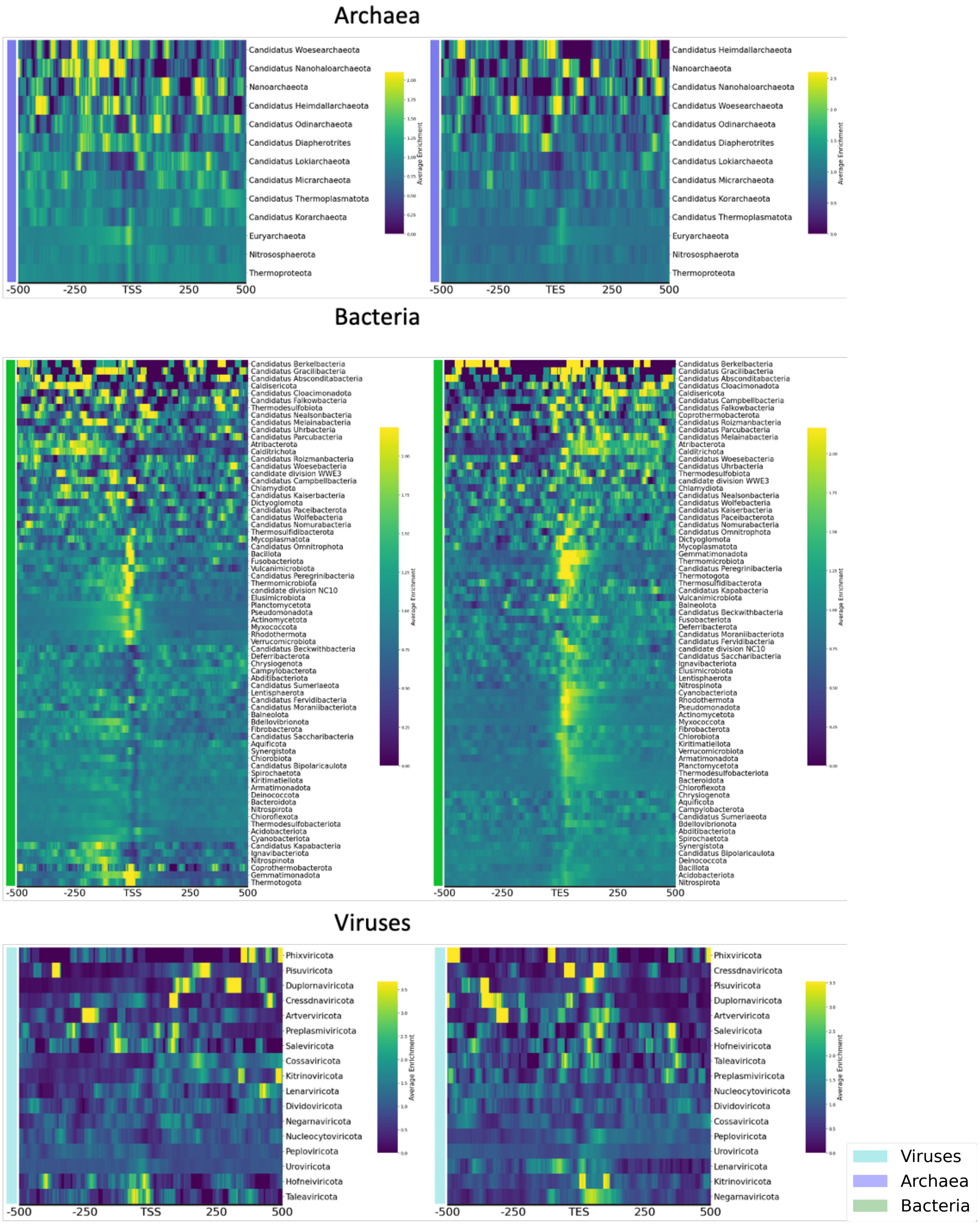


**Supplemental Figure 8: Distribution of G4s relative to transcription start and transcription end sites across archaeal, bacterial, and viral phyla.** Results shown from the G4Hunter-based algorithm.

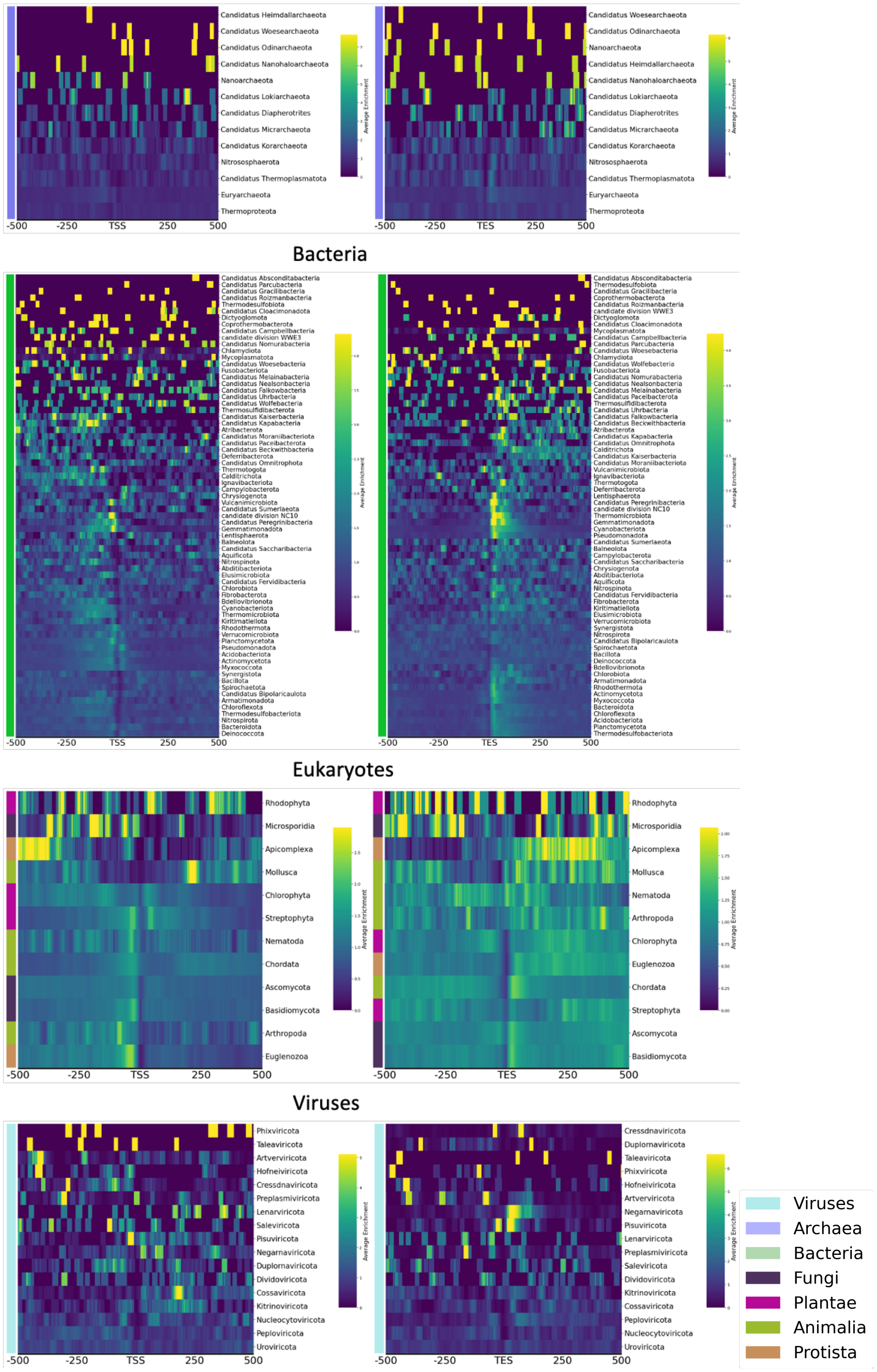


**Supplemental Figure 9: Distribution of G4s relative to transcription start and transcription end sites across archaeal, bacterial, and viral phyla.** Results shown for G4 motifs derived from the regex algorithm from the regex algorithm.

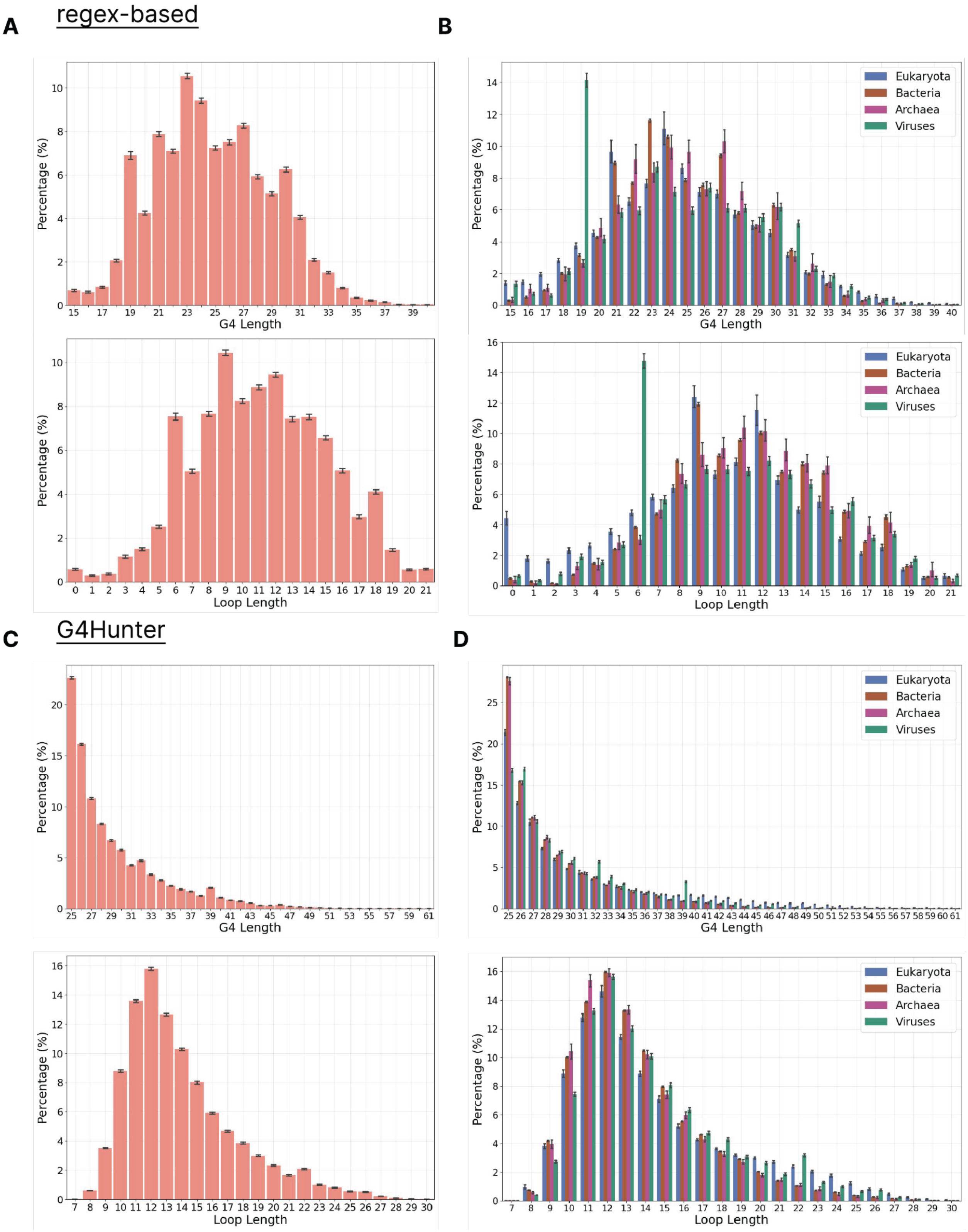


**Supplemental Figure 10: Length distribution of G4 lengths and of intervening loops of G4s across taxonomic groups. A-B)** Distribution of G-quadruplex sequence length and of intervening loops for the regex algorithm and the G4Hunter algorithm **C-D)** Distribution of G-

quadruplex total lengths and loop length for the three domains of life and viruses using the G4Hunter-based algorithm. Error bars represent the standard error.

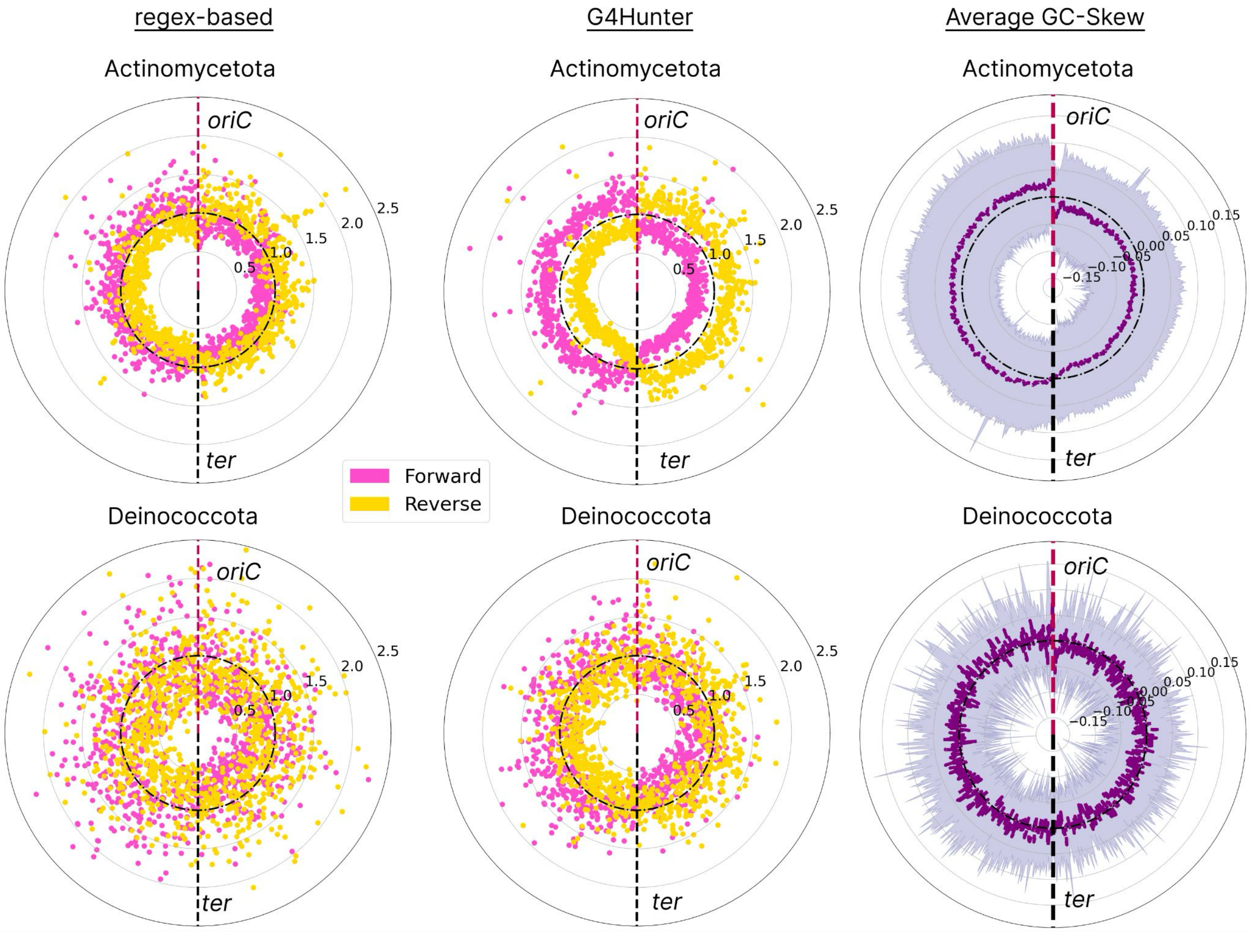


**Supplemental Figure 11: G4 distribution patterns relative to replication origin in bacterial phyla.** Results shown for Actionomycetota and Deinococcota. G4s in forward and reverse strand orientation are shown in yellow and pink, respectively. Results shown for G4Hunter-based and regex-based algorithms. Average GC skew is also calculated and shown with purple.

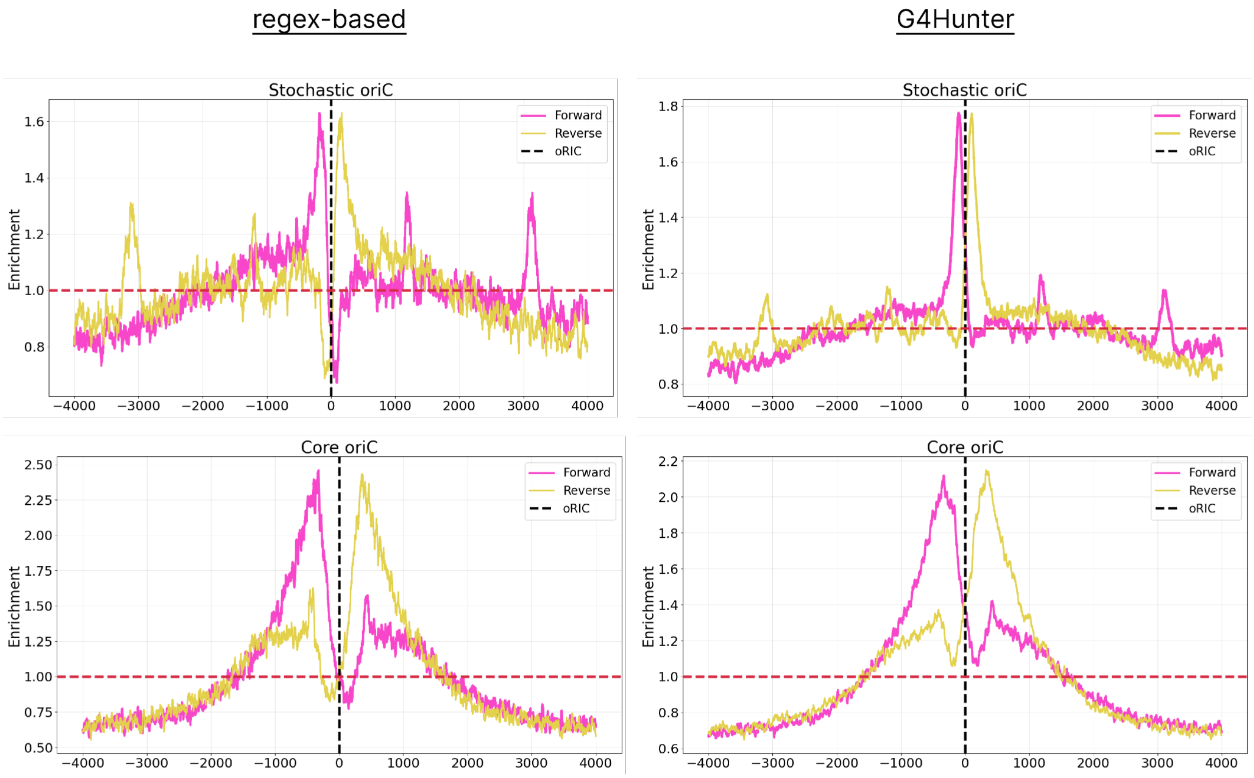


**Supplemental Figure 12: G4 distribution relative to origins of replication in *Homo sapiens*.** Distribution of G4s relative to the stochastic and core origins of replication for *Homo sapiens* around a window of kb from the origin of replication using both G4Hunter algorithm and the regex-based algorithm.

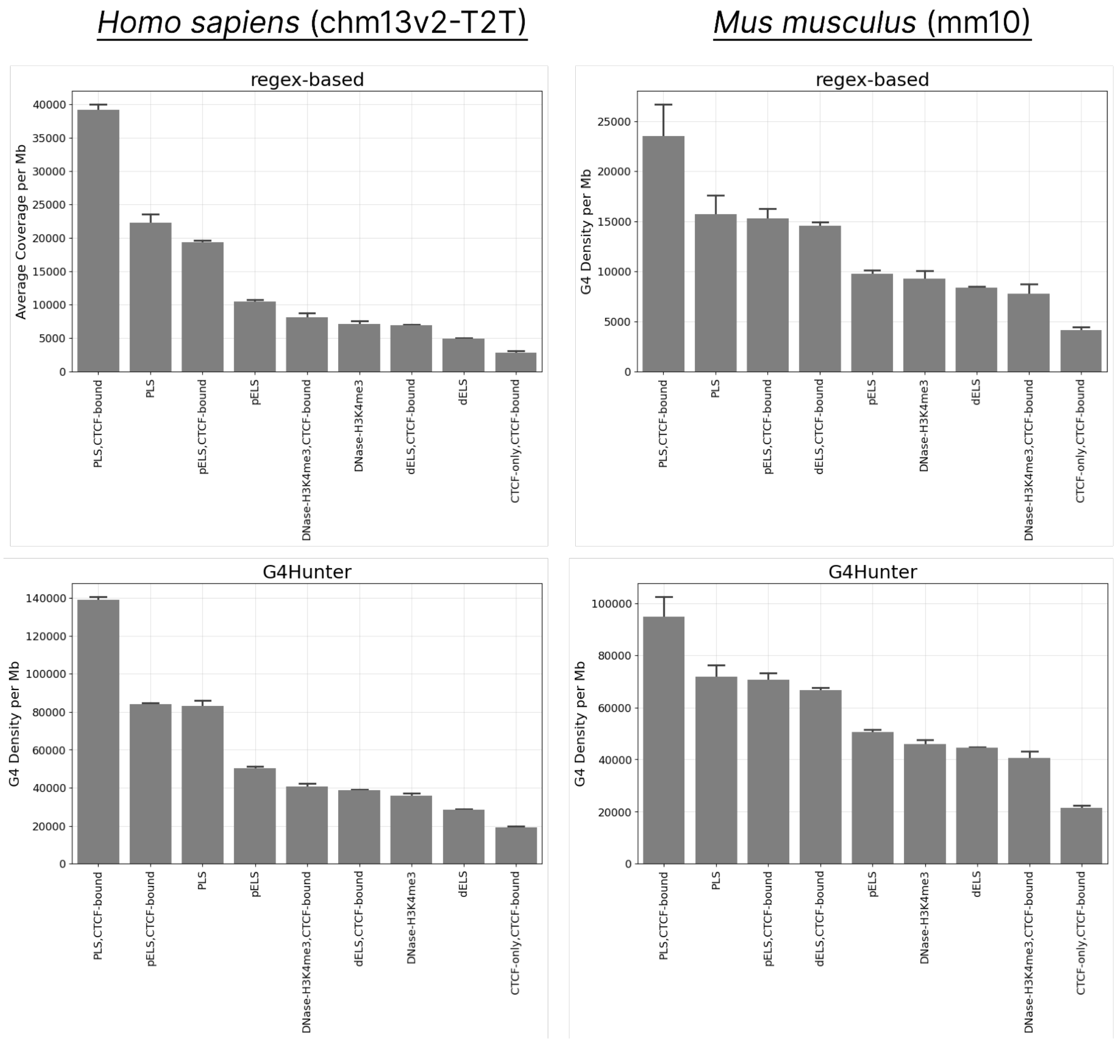


**Supplemental Figure 13: G4 distribution across cis-regulatory elements for *Homo sapiens* and *Mus musculus*.** Results shown display the distribution of G4 sequences across various cis-regulatory elements for *Homo sapiens* and *Mus musculus* using both G4Hunter algorithm and the regex algorithm.

**Supplemental Table 1: Average G4 motif densities**

**A. Average G4 motif density by domain (Data for Figure 2B)**

| Method | Domain | Average G4 Motif Density (per Mb) |
|---|---|---|
| Regex-based algorithm | Archaea | 26.41 |
| Regex-based algorithm | Bacteria | 29.56 |
| Regex-based algorithm | Eukaryota | 82.76 |
| Regex-based algorithm | Virus | 65.63 |
| G4Hunter algorithm | Archaea | 201.32 |
| G4Hunter algorithm | Bacteria | 147.82 |
| G4Hunter algorithm | Eukaryota | 327.04 |
| G4Hunter algorithm | Virus | 262.54 |

**B. Average G4 motif density in viruses by host domain.**

| Method | Host Domain | Average G4 Motif Density (per Mb) |
|---|---|---|
| Regex-based algorithm | Eukaryotic | 114 |
| Regex-based algorithm | Non-Eukaryotic | 29.49 |
| G4Hunter algorithm | Eukaryotic | 405.8 |
| G4Hunter algorithm | Non-Eukaryotic | 141.65 |

**C. Average G4 motif density in viruses by host domain for results based on G4s from G4Hunter.**

| Phylum | Average G4 Motif Density (per Mb) |
|---|---|
| Deinococcota | 2284.82 |
| Candidatus Bipolaricaulota | 1836.32 |
| Chlorophyta | 1382.03 |

| | |
|---|---|
| Peploviricota | 1335.95 |
| Euglenozoa | 1112.95 |
| Dividoviricota | 971.79 |
| Rhodothermota | 759.95 |
| Candidatus Korarchaeota | 742.42 |
| Cressdnaviricota | 703.71 |
| Myxococcota | 665.38 |
| Candidatus Verstraetearchaeota | 655.97 |
| Preplasmiviricota | 575.64 |
| Gemmatimonadota | 553.86 |
| Chordata | 532.45 |
| Synergistota | 507.41 |
| Basidiomycota | 492.67 |
| Candidatus Beckwithbacteria | 490.53 |
| Actinomycetota | 474.29 |
| Candidatus Binatota | 455.43 |
| Verrucomicrobiota | 450.42 |
| Armatimonadota | 442.03 |
| Chloroflexota | 439.41 |
| Microsporidia | 437.9 |
| Acidobacteriota | 435.66 |
| Thermodesulfobacteriota | 425.28 |
| candidate division NC10 | 424.66 |
| Kiritimatiellota | 421.14 |

| | |
|---|---|
| Thermomicrobiota | 420.15 |
| Cyanobacteriota | 406.92 |
| Artverviricota | 382.25 |
| Candidatus Latescibacteria | 379.82 |
| Cossaviricota | 363.86 |
| Kitrinoviricota | 363.13 |
| Nitrospirota | 351.15 |
| Planctomycetota | 349.26 |
| Abditibacteriota | 337.96 |
| Chrysiogenota | 334.3 |
| Mollusca | 330.3 |
| Candidatus Peregrinibacteria | 322.59 |
| Candidatus Tectomicrobia | 320.2 |
| Candidatus Fervidibacteria | 312.13 |
| Candidatus Sumerlaeota | 308.6 |
| Annelida | 279.17 |
| Nitrospinota | 272.9 |
| Saleviricota | 260.31 |
| Thermoproteota | 260.05 |
| Pisuviricota | 247.37 |
| Ascomycota | 243.85 |
| Hofneiviricota | 233.7 |
| Balneolota | 219.6 |

**D. Average G4 motif density in viruses by host domain for results based on the regex-based algorithm.**

| Phylum | Average G4 Motif Density (per Mb) |
|---|---|
| Deinococcota | 619.76 |
| Peploviricota | 566.73 |
| Chlorophyta | 518.64 |
| Candidatus Bipolaricaulota | 333.71 |
| Euglenozoa | 284.06 |
| Cressdnaviricota | 235 |
| Dividoviricota | 227.6 |
| Chordata | 174.13 |
| Myxococcota | 164.47 |
| Rhodothermota | 144.22 |
| Gemmatimonadota | 143.85 |
| Mollusca | 133.51 |
| Actinomycetota | 131.71 |
| Basidiomycota | 113.09 |
| Acidobacteriota | 108.65 |
| Candidatus Binatota | 107.37 |
| Candidatus Fervidibacteria | 94.77 |
| Saleviricota | 94.22 |
| Thermomicrobiota | 89.11 |
| Chloroflexota | 87.12 |
| candidate division NC10 | 85.37 |

| | |
|---|---|
| Preplasmiviricota | 84.26 |
| Armatimonadota | 82.21 |
| Candidatus Verstraetearchaeota | 81.92 |
| Nematomorpha | 79.35 |
| Cyanobacteriota | 78.49 |
| Thermodesulfobacteriota | 75.66 |
| Synergistota | 72.37 |
| Annelida | 71.6 |
| Planctomycetota | 68.82 |
| Candidatus Peregrinibacteria | 68.48 |
| Candidatus Korarchaeota | 66.64 |
| Verrucomicrobiota | 63.08 |
| Cossaviricota | 62.31 |
| Kiritimatiellota | 61.71 |
| Candidatus Tectomicrobia | 60.22 |
| Artverviricota | 59.69 |
| Candidatus Latescibacteria | 59.15 |
| Microsporidia | 54.56 |
| Nitrospirota | 53.48 |
| Heterolobosea | 50.62 |
| Kitrinoviricota | 50.39 |
| Chrysiogenota | 49.82 |
| Ascomycota | 49.68 |
| Streptophyta | 49.49 |

| | |
|---|---|
| Candidatus Beckwithbacteria | 48.58 |
| Abditibacteriota | 42.22 |
| Nitrospinota | 40.18 |
| Balneolota | 35.4 |
| Arthropoda | 35.15 |

**Supplemental Table 2: Phyla and host of the top 100 viral genomes having the highest G4 motif densities.**

**A.**

| Phylum | No. of Species | Total no. of G4s | Average G4 Motif Density (per Mb) | Hosts |
|---|---|---|---|---|
| Peploviricota | 73 | 38836 | 3489.00 | human, vertebrates |
| Kitrinoviricota | 11 | 384 | 4905.59 | fungi, land plants |
| Pisuviricota | 8 | 244 | 3731.96 | human, vertebrates |
| Artverviricota | 5 | 154 | 3463.75 | human, vertebrates |
| Dividoviricota | 1 | 63 | 3698.05 | |
| Duplornaviricota | 1 | 20 | 3801.56 | vertebrates, invertebrates |
| Preplasmiviricota | 1 | 1 | 3676.47 | |

**B.**

| Phylum | No. of Species | Total no. of G4s | Average G4 Motif Density (per Mb) | Hosts |
|---|---|---|---|---|
| Peploviricota | 94 | 38836 | 3489.00 | human, vertebrates |
| Kitrinoviricota | 4 | 384 | 4905.59 | land plants |
| Cressdnaviricota | 2 | 244 | 3731.96 | eukaryotic algae, invertebrates, vertebrates |

**Supplemental Table 3. Selected sequences from the G4 database.**

| Type | Seq (5′ to 3′) | Length (bp) |
|---|---|---|
| regex-based | GGGAAGGGGAGCCGTGGGGTAAAGAAGGG | 29 |
| regex-based | GGGGAGTTGGGGGAATAAGGGCGGAGGG | 28 |
| regex-based | GGGCTCGGGCTCGGGCTCGGG | 21 |
| regex-based | GGGTGCGGGTGCGGGTGCGGG | 21 |
| regex-based | GGGATGGGGGCGGGGGCGGG | 20 |
| G4Hunter | GGGCTGGGGCGGCTGGTGGTTCTGGG | 26 |
| G4Hunter | AGGTTGGTGTGGTGATTGGTGGGGGT | 26 |
